# Looking for Razors and Needles in a Haystack: Multifaceted Analysis of Suicidal Declarations on Social Media—A Pragmalinguistic Approach

**DOI:** 10.3390/ijerph182211759

**Published:** 2021-11-09

**Authors:** Michal Ptaszynski, Monika Zasko-Zielinska, Michal Marcinczuk, Gniewosz Leliwa, Marcin Fortuna, Kamil Soliwoda, Ida Dziublewska, Olimpia Hubert, Pawel Skrzek, Jan Piesiewicz, Paula Karbowska, Maria Dowgiallo, Juuso Eronen, Patrycja Tempska, Maciej Brochocki, Marek Godny, Michal Wroczynski

**Affiliations:** 1 Department of Computer Science, Kitami Institute of Technology, Kitami 090-8507, Japan; eronen.juuso@gmail.com; 2 Department of Contemporary Polish Language, Faculty of Philology, University of Wrocław, 50-140 Wrocław, Poland; mzaskozielinska@gmail.com; 3 Samurai Labs, 81-824 Sopot, Poland; michal.marcinczuk@samurailabs.ai (M.M.); gniewosz.leliwa@samurailabs.ai (G.L.); marcin.fortuna@samurailabs.ai (M.F.); kamil.soliwoda@samurailabs.ai (K.S.); ida.dziublewska@samurailabs.ai (I.D.); olimpia.hubert@gmail.com (O.H.); Pawel.m.skrzek@gmail.com (P.S.); jan.piesiewicz@samurailabs.ai (J.P.); paula.karbowska@samurailabs.ai (P.K.); mdowgiallo1@st.swps.edu.pl (M.D.); patrycja@samurailabs.ai (P.T.); maciej.brochocki@samurailabs.ai (M.B.); marek.bobcow@samurailabs.ai (M.G.); michal.wroczynski@samurailabs.ai (M.W.); 4 Department of Computational Intelligence, Faculty of Computer Science and Management, Wrocław University of Science and Technology, 50-370 Wrocław, Poland; 5 Institute of English and American Studies, Glottodidactics and Natural Language Processing Division, University of Gdańsk, 80-308 Gdańsk, Poland; 6 Institute of Clinical Psychology, SWPS University of Social Sciences and Humanities, 03-815 Warsaw, Poland

**Keywords:** suicidal declarations, LIWC, social media

## Abstract

In this paper, we study language used by suicidal users on Reddit social media platform. To do that, we firstly collect a large-scale dataset of Reddit posts and annotate it with highly trained and expert annotators under a rigorous annotation scheme. Next, we perform a multifaceted analysis of the dataset, including: (1) the analysis of user activity before and after posting a suicidal message, and (2) a pragmalinguistic study on the vocabulary used by suicidal users. In the second part of the analysis, we apply LIWC, a dictionary-based toolset widely used in psychology and linguistic research, which provides a wide range of linguistic category annotations on text. However, since raw LIWC scores are not sufficiently reliable, or informative, we propose a procedure to decrease the possibility of unreliable and misleading LIWC scores leading to misleading conclusions by analyzing not each category separately, but in pairs with other categories. The analysis of the results supported the validity of the proposed approach by revealing a number of valuable information on the vocabulary used by suicidal users and helped to pin-point false predictors. For example, we were able to specify that death-related words, typically associated with suicidal posts in the majority of the literature, become false predictors, when they co-occur with apostrophes, even in high-risk subreddits. On the other hand, the category-pair based disambiguation helped to specify that death becomes a predictor only when co-occurring with future-focused language, informal language, discrepancy, or 1st person pronouns. The promising applicability of the approach was additionally analyzed for its limitations, where we found out that although LIWC is a useful and easily applicable tool, the lack of any contextual processing makes it unsuitable for application in psychological and linguistic studies. We conclude that disadvantages of LIWC can be easily overcome by creating a number of high-performance AI-based classifiers trained for annotation of similar categories as LIWC, which we plan to pursue in future work.

## 1. Introduction

Suicide is one of the global leading causes of death [1], with about eight hundred thousand people taking their own lives every year [2]. However, compared to other purely biological and external common causes of death, including cancer and cardiovascular diseases, preconditions leading to suicides (e.g., depression, schizophrenia, hopelessness, PTSD, etc.) are notoriously difficult to diagnose, while the occurrence of the event of suicide itself is much more problematic to predict, which makes the problem of suicides a comparatively much more complex problem to solve on a global scale. Moreover, most of the other biological or natural common causes of death, such as cardiovascular diseases or cancer have reached a relatively high and growing social awareness [3,4,5,6,7,8,9], with well-funded research infrastructure(https://report.nih.gov/funding/categorical-spending#/, accessed on 2 November 2021) and a world-wide push to develop medical treatments or vaccines [10,11]. On the other hand, other human-inflicted causes of death, such as road injuries, which mostly result from direct conscious human activity, could be decreased by raising awareness and educating the population to reach higher levels of responsibility, e.g., regarding road behavior [12].

Differently to the above, suicide and its causes (e.g., depression or schizophrenia, etc.) have grown in global society, largely unnoticed for many years [13,14,15]. In 2017 alone, 1.4% of global deaths were from suicide, while in some countries, this share was as high as 5% (https://ourworldindata.org/causes-of-death, accessed on 2 November 2021). The statistics indicate that the global yearly number of suicide victims, or about eight hundred thousand cases, is twice as much as for homicide victims (https://ourworldindata.org/suicide, accessed on 2 November 2021). Even more troubling statistics show that suicide is one of the leading causes of death in young people (https://ourworldindata.org/grapher/suicide-deaths-by-age, accessed on 2 November 2021).

The above-mentioned situation has been further exacerbated during the COVID-19 pandemic, with a sudden increase in cases of depression and suicide all over the world [16,17,18,19,20,21,22,23,24,25,26,27]. With the aftermath of the COVID-19 pandemic, presumably to last for several years even after the end of the pandemic itself in various areas of everyday life, beginning with the economy [28], tourism [29] as well as in the area of global mental health [30], it becomes clear that more decisive and concrete measures need to be taken to effectively and efficiently detect and diagnose suicidal tendencies, and prevent the suicides in the global population.

Studies show that with proper significant systemic effort, suicidal intent can be detected in time [31] and suicide can be prevented using psychological risk assessment tools [32], improvement of policies [33], or extensive training of personnel [34]. Unfortunately, all those measures are time consuming and difficult to implement and execute, which along with the low social awareness of the problem of suicide, hinder early detection of potential suicide attempts. The detection of a suicidal attempt is also hindered by the typical procedural complexity of the process during which the detection can take place, namely, during consultations with mental health specialists. Those consultations often need to be numerous for the specialists to be able to collect sufficient information about the patient and develop their psychological profile. Moreover, often such psychological or clinical profiles are also not extensive enough, which is only revealed in a farewell letter or a suicide note [35].

To improve and support the process of early detection of potential suicide victims, recent cross-disciplinary studies proposed a novel path to less invasive and more robust suicide intent detection, namely, by using information and communication technologies (ICT) to monitor social network services (SNS), such as Facebook (https://www.facebook.com/, accessed on 2 November 2021), Twitter (https://twitter.com/, accessed on 2 November 2021), or Reddit (https://www.reddit.com/, accessed on 2 November 2021) and analysing users’ mental health based on their activity and messages with the use of artificial intelligence (AI) algorithms [36,37,38,39,40]. By definition, such monitoring is not aimed at fully solving the the problem of suicide, nor is it aimed at displacing actual mental health specialists, but rather could work as an initial triage that could help the specialists reach out to potential suicide victims more efficiently and aid them with proper psychological support at an early stage. Especially, Shing et al. (2018) [39] and Zirikly et al. (2019) [40] argue that, since the victims of suicides are often young people, and most young people also use various internet communication means, such as SNS, it is possible that (1) the young people consciously, or subconsciously express their suicidal intents in their Internet messages, and (2) due to being digital natives [41], would be willing to answer a proactive step made towards them. Moreover, such methods, when developed and executed properly, could not only be well suited to aid young Internet users, but also with the current age of information explosion [42] could shortly become necessary and one of the few realistic means to reach out to the young people in need [43,44]. Such expectations are supported by the fact that AI-based methods have been applied in various areas of medicine more and more often, proving that AI, when utilized with responsibility and within proper ethical standards has a wide potential to help doctors and health care practitioners in their hard work [45,46,47,48,49,50,51,52].

In this paper we contribute to the described above newly developed field, usually called *suicidal intent detection* or *suicidal declaration detection*. Suicidal intent detection is a new multidisciplinary research field combining such related fields as psychology, data science, and natural language processing with the aim to provide help and support to suicidal individuals. The development of this field was made possible thanks to the increase in wide availability of open access public communication taking place online. Until recently, the decision to commit suicide has been made exclusively in the mind of an individual or disclosed only to the closest friends and relatives. Researchers had a chance to gather data on the process of emergence of suicidal ideation only after a person died. Today, complex analyses of big data coupled with interdisciplinary cooperation between psychologists, especially suicide experts, linguists, and information technology professionals allow quicker interventions and strongly facilitate providing help and support.

In our research, we make an initial study of the problem of suicidal intent being revealed online. We take the data science approach supported with statistical analysis of a large dataset containing suicidal declarations of users on Reddit, one of the most popular SNS platforms, widely known for its freedom of speech. The statistical analysis helps us understand the phenomenon and find meaningful correlations both within user activity as well as in the vocabulary used in such declarations.

### 1.1. Research Motivation and Contributions

The motivation for this research was the following. Although research on the language of suicidal individuals is a well established field, an overwhelming majority of the research is based either on interview records with mental health patients who later committed suicide, or on farewell letters written by suicide victims. On the other hand, the internet, especially social media such as Reddit, provides a sufficiently anonymous space where an increasingly large numbers of such individuals have been venting their stress and concerns. This opens an opportunity for counselors and suicide experts to efficiently reach such individuals and help them before the tragedy happens. Unfortunately, the practical problem in facilitating such help is in filtering out messages that only resemble suicidal messages, but are in fact written by not suicidal users (e.g., overwhelming use of death related vocabulary on gaming channels). This motivated us to collect a large scale suicidal message dataset and study it thoroughly to find clear characteristics of suicidal messages, which could be applicable as guidelines for suicide experts and counselors. In this regard we performed the following research, of which the most straightforward contributions are the following:

- Specified four groups of subreddits with different intensity of suicidal messages;
- Created a symbolic system for spotting suicidal messages;
- Created a corpus of suicidal and seemingly suicidal posts from the Reddit social network;
- Created an expert-based annotation scheme and guidelines for annotations of suicidal datasets;
- Created Reddit Suicidal Query (RSQ) Dataset by annotating the corpus with the help of suicide experts and highly trained annotators;
- Analyzed the dataset thoroughly to confirm and improve knowledge base on suicidal language (first so thorough analysis of suicidal messages on this scale);
- Created an approach for analysis of suicidal messages by using correlating with one another the results of Linguistic Inquiry with Word Count (LIWC), which allows to minimize LIWC errors and retains only the most informative results;
- Collected a number of category pairs with the strongest correlations in each risk group which can be directly applied by suicide experts and counselors to triage through unrelated contents;
- Specified limitations of applicability of LIWC—a tool widely used in psychology—in suicide research, and outlined how to improve its lack of contextual processing by developing a number of context-aware machine learning classifiers;
- Prepared grounds for further training of machine learning classifiers to automatically differentiate between actually suicidal and pseudo suicidal messages.

### 1.2. Paper Outline

The remainder of the paper is as follows. Section 2 describes our field study of previous research conducted in linguistic analysis of suicidal and presuicidal discourse in general (Section 2.1), suicidal declarations in presuicidal discourse in particular (Section 2.2), availability of corpus data and other Internet-based resources of suicidal text, as well as previous machine learning-based studies in suicidal text detection (Section 2.3). In Section 3, we describe how the dataset used in this research was collected and developed. We also briefly describe the technology applied in automatic suicidal text detection (Section 3.1). Next, we perform a statistical analysis of the dataset to gain new insights on suicidal declarations expressed online (Section 4). In particular, we analyse the tendencies found in user activity online (Section 4.2), and analyse correlations in categories of the used vocabulary (Section 4.3). Finally, we provide a thorough general summarizing discussion of the whole study (Section 5), in which we acknowledge several limitations of this study (Section 5.2) as well as discuss ethical concerns that should be taken into account in research similar to ours (Section 5.3). Lastly, we conclude the paper and draw a number of possible future directions in Section 6.

## 2. Previous Research

### 2.1. Suicidal and Presuicidal Discourse

Suicidal discourse is a type of discourse focused on suicide and taking place among various interlocutors across various genres. It can be issued by both suicidal individuals as well as persons touching upon suicidal topics for scientific, journalistic, artistic, and therapeutic reasons ([53], p. 156).

In contrast, presuicidal discourse refers to the utterances made by one person at various stages of suicidal ideation development. Suicidal ideation may either be reverted to the previous state or lead to making a decision to take one’s own life ([54], p. 272). In line with narration theory, presuicidal discourse aims at putting the experiences of an individual in order within the framework of the narration about one’s own life constructed over an extended period of time ([55], p. 414). It is not easy to make observations on presuicidal discourse as a whole, since it is directed at a wide range of recipients and audiences, with the only reoccurring participant being the source. Access to utterances of this kind is very limited, as they include ephemeral spoken acts and personal documents, which are typically stored as part of private property, unless communication takes place on the internet. The stage of presuicidal discourse that is the easiest to identify is the last stage, since its symptoms are most conspicuous. However, it is usually identified only during an investigation of an individual’s life after their suicidal act or attempt. presuicidal discourse subsumes primarily: suicide notes [56], diaries [57,58], letters left by individuals who committed suicide ([59], pp. 671–678); ([58], pp. 121–128), blogs [60], e-mails [61], and text messages (SMS) ([62], pp. 1412–1430) written by patients in therapy, and recorded interviews with suicide attempters ([57], pp. 140–144). What is much less straightforward as part of the presuicidal discourse is depressive periods of silence, cry for help, or literary texts with suicidal content. Presuicidal communication includes both spoken and written content, conveyed through a wide range of channels. Due to culturally and socially conditioned changes in this type of discourse, the role of computer-mediated communication (the internet, mobile phones, etc.) is increasing ([63], pp. 378–394). Even suicide letters, traditionally written by hand, are now usually typed ([64], pp. 1379–1394). This way, the potential committer may substitute the possibility of a dialogue with an utterance directed at a predefined and usually anonymous audience by, for example, recording a video, writing an Internet forum post [65] or a Twitter post [66,67]. With the emergence of social media, the size of the audience and the number of recipients of presuicidal discourse has significantly risen [68], which results in a larger number of professionals being able to provide instant help to suicidal individuals.

### 2.2. Suicidal Declarations in Presuicidal Discourse

The texts realizing the presuicidal discourse can belong to different genres, which reflect various life stages of a given individual. Some of them can extend over long periods, for example a diary, called by Leenars ‘a suicide note with history’ ([69], p. 37). They can also include numerous suicidal declarations with a varying degree of certainty.

The most thoroughly researched type of text in presuicidal discourse is the suicide note. It has been identified what speech acts are typically expressed by the author [70,71] and the rhetorical moves (or: discursive moves) characteristic for such texts ([72], pp. 88–101). The knowledge gained from the analysis of the structure of suicide notes may be very useful while describing other types of texts belonging to presuicidal discourse. Nevertheless, while assessing the risk of potential suicide threat, it is crucial to take into account the genre of the previous messages on the axis of time in a given individual’s discourse, so that other people can witness this communication and provide help and support.

Suicidal declaration is defined as information about the decision to take one’s own life. It is often verbalized by suicidal individuals, albeit not always in a written form ([73], pp. 2239–2253). It is not an indispensable part of a suicide note due to the situational context. When the dead body of a suicide committer is found, no declaration is necessary. Instead, a suicide note typically includes an explanation of the decision, described in the rhetorical structure of the genre as consisting of *moves* and *steps* ([72], p. 92):

“2 Providing explanation

  2.1 Providing larger context or background
  2.2 Giving reason(s) or justifying
  2.3 Taking responsibility or ascribing responsibility to others
  2.4 Expressing feelings about act”

Since pragmatic conditions strongly impact the perception of a given genre, the structure and detailed objectives of a suicidal declaration can be influenced by the recipients, the audience, or the channel through which the message is conveyed. A suicidal declaration posted to a specific internet forum is inextricably connected with the pragmatic situation characteristic for this anonymous community. The maximal structure of the declaration can contain elements which also appear in other psychological concepts on suicidal risk assessment, based upon, for example, interviews with patients: Suicide Ideation Scale [74,75] and Suicide Intent Scale ([76], pp. 80–87). These elements include information about a death wish and its duration (suicidal thoughts), information about readiness to take one’s own life, information about the ultimate lethal intent, explanation of one’s reasons for this decision, the intended suicide method, information about the current deterrents of active attempt, and information about the current state of preparation (e.g., a suicide note, a final will).

### 2.3. Internet-Based Resources for Suicidal Text Analysis

The first and the most frequently quoted corpus of suicidal texts was collected by Shneidman and Farberow, which consists of 33 genuine suicide notes and 33 simulated ones, adjusted on the basis of sender data [56]. The earliest electronic materials used for research on suicidal texts are collections of written documents, which were originally handwritten and scanned, obtaining the electronic format after transcription, e.g., Suicide Note Corpus [77] or Polish Corpus of Suicide Notes [78]. Suicidal texts were collected for various purposes. Apart from the classical idea of exploring a suicidal mind, a number of corpora were created to analyze changes taking place in the language of suicidal individuals ([79], pp. 133–138) to indicate differences between genuine and fabricated suicide notes for the purposes of forensic linguistics [80]. However, the main aim of collecting suicidal text corpora based on online resources is detecting suicidal declarations with a goal to prevent possible suicidal attempts. Researchers use, e.g., the resources of Twitter ([81], pp. 183–188), blogs (RusSuiCorpus by Litvinova et al. (2018) [82]) or forum posts, such as Reddit [83]. The availability of research material and the abundance of texts allows for using a wide range of analytical tools at a previously unprecedented scale: it is possible to combine manual annotation, statistics, machine learning techniques, and sentiment analysis. One of the biggest disadvantages of big data studies is the shortage of metadata on the message senders. This is why the data collected online are often paired with medical sources [84]. It is, however, possible to conduct large-scale comparisons of suicidal discourse data, for instance, between presuicidal and nonsuicidal posts. The findings of the earlier research on suicide notes can thus be verified and supported by Natural Language Processing (NLP) tools, as it is shown, for example for the *Corpus of Emotion Annotated Suicide Notes in English* ([85], pp. 1618–1626). NLP makes it possible to process and analyse large amounts of data in real time. Nonetheless, the attempts to detect suicidal declarations in social media are directed at a genre linguistically different from typical suicide notes. Texts collected from online forums and social media have a discourse characteristics and pragmatic structure different from a typical suicide note.

## 3. Development of Samurai Labs Reddit Suicidal Query (RSQ) Dataset and Related Technology

### 3.1. Technology Applied in Initial Automatic Suicidal Text Detection

An important part of the project was developing a symbolic rule-based system for initial detection of suicidal texts.

The system was based on Samurai, a technology developed previously by Samurai Labs (https://www.samurailabs.ai/, accessed on 2 November 2021), and described in Ptaszynski et al. (2018) [86] and later in Wroczynski et al. (2021) [87] The technology, which was initially developed for cyberbullying detection and in this project adapted to detect only suicidal texts, comprises a combination of symbolic and statistical methods, where each statistical component (e.g., a machine learning model) is governed by a symbolic component utilizing a variety of natural language processing methods (e.g., tokenization, syntactic parsing, etc.). Symbolic components are used to determine if a potentially relevant utterance is not a part of a broader utterance indicating that the first one should not be considered relevant. For example, an utterance “you are an idiot” (We explain the technology using examples not related to suicidal texts) is potentially abusive, but it in fact is not, if it appears as a part of a longer utterance such as “I cannot believe he said you are an idiot”. Another example of using symbolic components is determining if a phrase is targeted at self (e.g., using a linking verb to assign the phrase with a second person as in the “you are an idiot” example).

Samurai [87] employs a compositional approach, where each problem is divided into a set of corresponding sub-problems represented with language phenomena (e.g., speech acts), and detected independently using highly precise contextual models. For example, suicidal texts comprise a high-level category that can be divided into specific language phenomena (mid-level categories) such as Suicidal Thoughts, Plan Method, or Cry For Help, or other categories mentioned in Section 3.3. Symbolic rules for the version of Samurai covering suicidal text detection were developed along with the processes of data collection and annotation.

**Figure 1:**
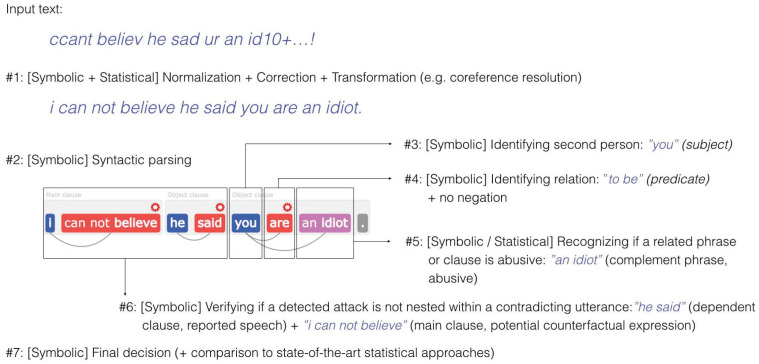
Example of processing of one sentence by the applied Samurai technology.

Figure 1 illustrates how the input text (“ccant believ he sad ur an id10+…!”) is processed step-by-step utilizing both statistical and symbolic methods.

In practice, this means that a whole variety of constructions can be detected without the need of constructing a fixed list of dictionary words defined *a priori*. Due to utilizing symbolic components that oversee statistical components, {Samurai} recognizes complex linguistic phenomena (such as indirect speech, rhetorical figures or counter-factual expressions) to distinguish suicidal phrases used in suicidal context from other, e.g., gaming context, greatly reducing the number of false alarms.

### 3.2. Data Collection

The dataset of Reddit posts used for this research, as well as later for the development of a symbolic system for the detection of suicidal texts, and training of machine learning algorithms, and testing their effectiveness has been developed over roughly half a year. Along with the collection and annotations of the new parts of data, we started developing a system based on a set of symbolic rules to detect phrases and sentence patterns typical for suicidal texts.

The first steps of symbolic system development (which took place in December 2019) involved collecting our first initial corpus, which was still small-scale and unstructured. We decided to build the dataset based on Reddit (https://www.reddit.com/, accessed on 2 November 2021) following previous works [39]. We performed a Reddit data dump using the official Reddit API and subjected it to a keyword-based search to find potential suicidal submissions (the keyword list included such items as ‘suicide’, ‘suicidal’ or ‘kill myself’). We collected ca. 500 submissions from r/SuicideWatch, another 500 from r/suicidalthoughts and yet another 500 from r/depression as potential True Positives. To find potential False Positives, we performed a search on selected subreddits which we subjectively judged to contain a lot of controversial content and touching upon sensitive topics: r/MGTOW, r/askgaybros, r/Christianity, r/politics, r/Feminism, r/AskReddit, r/AskMen, r/MURICA, r/teenagers, r/DebateReligion, r/relationship_advice, r/KotakuInAction, r/askwomenadvice, r/soccer, r/AskWomen, r/4chan, r/atheism, r/ Libertarian, r/BlackPeopleTwitter, r/insanepeoplefacebook, r/AntiVegan, r/unpopularopinion, r/ Showerthoughts, r/ZeroWaste, r/worldnews, r/guns, r/The_Donald, r/Catholicism, and r/GenderCritical. This way, we managed to collect 1750 possible False Positives. These data, consisting of 1500 potential True Positives and 1750 False Positives, served as a basis for building the first system rules, which laid the foundation of all subsequent work.

In order to test the newly created system prototype, in January 2020 we downloaded ca. 4.8m Reddit comments and ran first tests. We obtained ca. 19k matches from r/SuicideWatch, which later provided a framework for refining and expanding the existing symbolic rules.

At the beginning of February 2020, we became aware of the significant over-classification of the system and made first attempts to curb the number of rules generating too many False Positives. At that time there arose a need to apply a machine learning (ML) model which would operate on the output of the symbolic module and filter out all non-suicidal matches (which included such sentences as *‘OMG lol I want to die!!!’*, or *‘I am so embarrassed, why can’t I just drop dead already’*.). This allowed us to develop a hybrid artificial intelligence (AI) approach combining both symbolic rule-based detection and ML-based classification. In the first step, we created a small corpus consisting of 100 True Positives, 100 rhetorical False Positives (i.e., submissions including suicide-related expressions used as a rhetorical figure, e.g., *‘How did you guys stay focused on work? I want to die right now.’*) and 100 False Positives referring to a suicide of a third person (rather than the author of the submission themselves, e.g., *’My friend is heavily depressed and suicidal’*). We conducted a pilot study, testing various ML methods, but the results were not satisfying at that initial and mostly exploratory stage.

We continued to collect the data, which in February 2020 resulted in a collection of 1306 True Positives (from suicidal contexts, i.e., such subreddits as r/SuicideWatch, r/depression, and r/suicidalthoughts) and 1211 False Positives (mainly from gaming-related subreddits). Subsequently, a number of competing ML models were trained to eliminate the FPs found in the output of the symbolic system. However, a corpus of this size proved insufficient as well. The results were far from satisfying, with a large part of the problem being the gaming-related focus of the FP collection: a model trained on gaming data did not perform well on FPs gathered in other contexts. We discerned a need of introducing a more fine-grained division within the training set.

With these considerations in mind, we started distinguishing between ‘suicidal contexts’ (such as r/SuicideWatch or r/depression, in which users often submit suicidal messages), ‘grey areas’ (such as r/teenagers and r/offmychest, in which suicidal messages can sometimes occur), and gaming-related ones (in which actual suicidal messages occur rarely, or almost never, but messages resembling suicidal messages occur frequently, e.g., by someone reporting they are going to die in a game). For the purpose of collecting the improved dataset, at the end of March 2020 we extracted 2000 suicidal True Positives, 2000 ‘grey area’ True Positives, 2000 ‘grey area’ False Positives, and 2000 gaming False Positives. They formed our first somewhat reliable mid-size ‘4k+4k’ training set.

This decision was fruitful and brought a significant rise in the effectiveness of the ML filter. However, the aforementioned three-way division of data (suicidal, grey area, non-suicidal) still did not seem to be fine-grained enough to reflect the actual diversity of the data. The category of ‘grey areas’ turned to be very broad and to encompass highly varied patterns.

To ensure an even higher level of performance, we introduced a division of data sources into ‘levels of risk’, which referred to the relative probability that a given source includes suicidal content (similarly to Shing et al. (2018) [39]). The levels of risk encompassed:

- Suicidal (r/SuicideWatch, r/depression etc.);
- High (r/mentalhealth, r/BPD etc.);
- Medium (r/anxiety, r/offmychest, r/bipolar etc.);
- Low (gaming);
- Low (other non-gaming subreddits).

The division of the full list of subreddits into risk levels was based on both our own subjective experience with Reddit data, analysis of the focus of the subreddit as well as objective statistical observations, i.e., the numbers of matches from a given subreddit which turned out to be True Positives after annotation. The threshold to include a subreddit in each category was set as follows:

- equal and above 20% of suicidal messages → Suicidal
- between 10% and up to 20% → High
- between 5% and up to 10% → Medium
- below 5% → Low

The additional distinction between ‘gaming’ low and ‘non-gaming’ low groups was introduced for the sake of False Positives alone, since True Positives have always been nearly non-existent in the gaming contexts. See Appendix A for a full list of subreddits included in each group.

Since not all of these groups were sufficiently represented in the hitherto collected dataset, there arose a need to retrieve another batch of Reddit submissions. Shortly thereafter, we performed a download of full continuous Reddit data from the periods 9–16 March 2020 and 26 March–2 April 2020. Having run our symbolic systems on the data and annotated the results, in mid-April we had a whole new array of valuable data at our disposal. We proceeded to create the entirely new robust dataset. It included a part of the previous set as a seed, but was supplemented with the newly collected data. The new dataset consisted of almost 18k examples, divided into 4 TP categories (suicidal, high, medium, low) and 5 FP categories (suicidal, high, medium, non-gaming low, gaming low). This was the first prototype of the main dataset being used until now.

In the meantime, the team was progressively working on polishing and optimising the performance of the symbolic part of the system. Many False Positive matches were eliminated by corrections and patches applied to the symbolic rules. Crucially, we managed to eliminate almost all FPs referring to the third person and were left with almost exclusively rhetorical (and similar) FPs. At the same time, after consultations with suicide language expert, we improved our definition of ‘True Positive’, which resulted in reannotation of a sizable number of data items (and a revision of a number of symbolic rules). Most importantly, we replaced simple binary annotation (‘suicidal’ vs. ‘non-suicidal’) with a more complex annotation system based on identifying specific *speech acts/rhetorical moves* [70,71,72] in each submission (for more details, see Section 3.3). The core of the dataset remained mostly unchanged, with the exception of correction of a small number of annotation errors caught in the process of constant revision.

The detailed statistics of the data included in the dataset are presented in Table 1:

Unfortunately, having only the initial training dataset was not sufficient for our purposes—we also needed another corpus to test the effectiveness of newly trained ML models. Although the evaluation could be performed using cross-validation (using part of the whole dataset for training and the rest for testing and repeating the process until all parts are used in testing), since the data for the training dataset was collected in a close time range, its contents could be thematically similar to some extent, which could result in the overfitting of ML models to the data. Therefore, we decided to collect an additional smaller dataset purely for testing purposes. To fulfill this goal, we performed three iterations of Reddit data dump from the following periods: 13–19 April 2020, 4–10 May 2020, and more recently, 8–14 October 2020. The data from the former two sets were initially annotated in a binary manner in the period following the download, only to be reannotated through the lens of speech act-based categorisation in August 2020. The October dump was directly annotated with speech acts. Crucially, these three datasets contain fully continuous Reddit data and reflect the natural influx of submissions from Reddit in real time (unlike the training dataset, which contains data from various periods). This approach guaranteed that the tests performed on the corpus would be representative, unbiased, and would reliably correspond to the results obtained in production environments.

Each step in the development of the new dataset was accompanied by consecutive attempts to train an effective ML model to filter out False Positives. After dividing the data into the ‘risk levels’, a decision was made to train four (rather than one) ML models, each operating at a different risk level, to be deployed to analyse different subreddits. This strategy proved to be a success, as the submissions at each risk level displayed intra-level similarities which made it much easier for ML algorithms to generalize proper text features and to correctly distinguish between genuine suicide declarations and False Positives.

In summary, the whole process of corpus collection, annotation and internal classification was complicated and tedious, but it was indubitably of paramount importance to accomplishing the ultimate goal of the project.

### 3.3. Process of Annotation

At the beginning of the annotation we made several non-bendable assumptions for assure the quality of annotations. Firstly, the annotations would be performed only by experts, which includes psychology professionals (experts) and psychology PhD candidates with practice (near-experts). In addition, a provable high level of English language was a necessity. The annotation was conducted in a 2+1 annotation scheme according to clearly specified guidelines, with two annotators independently annotating provided samples (Reddit posts) and one super-annotator with the highest knowledge of both the annotated matter as well as the annotation process curating the annotations. In case of inconsistencies, the super-annotator would specify the correct annotation and reach out to both annotators to discuss with them their decisions regarding the annotations. Inter-annotator agreement was frequently measured between the annotators and between the annotators and the super-annotator to monitor the quality of annotation, and the annotators who were falling behind were additionally trained. The guidelines for annotations were constantly improved on each stage of annotation, with discussions being held frequently between the annotators, the super-annotator and suicide language experts. We always allowed the possibility that, even if an annotator disagrees with the super-annotator, the reasoning behind the annotator could be correct and could improve the annotation process and guidelines. The annotation was performed using WebAnno annotation tool (https://webanno.github.io/webanno/, accessed on 2 November 2021). After several steps of improving the annotation process, which was correlated with improving the data collection (see Section 3.2), we used the most matured version of the annotation process and guidelines until the end of the study.

The annotation was carried out by 7 annotators who were assigning the following ten categories of suicidal content to the provided Reddit posts:

- Being Burden;
- Complaint;
- Cry for Help;
- Declaration;
- Farewell;
- Past Attempts;
- Plan Method;
- Self Harm;
- Suicidal Thoughts;
- Traumatic Experiences.

The categories, which were selected after several discussions with psychologists and suicidal language experts, represent the traditional speech acts/rhetorical moves often used in psychology to analyse suicidal messages [70,71], and in this or similar form are a well established also in suicidal texts classification [39,40]. When it comes to the specific annotation procedures, the annotators annotated not only labels on the posts, but also annotated spans of phrases which are representing each category. This way every post could be annotated with a number of categories with specific information on its word span, which was helpful in the development of symbolic rules for automatic detection.

Throughout the process, some changes in the annotation instructions had to be introduced in order to improve the quality of annotated data. The categories Suicidal Thoughts, Declaration and Plan Method were eventually better defined as a gradual process finally leading to suicidal attempt, rather than individual occurrences. Furthermore, the definition of Complaint was narrowed down to the description of an individual’s mental state, such as mental disorders, pharmacotherapy, psychosomatic symptoms, numbness. Therefore not all instances of displeasure with oneself were considered. All the data annotated based on previous instructions were changed and reannotated according to new modifications.

The quality of annotated data was examined by an annotation expert. A random subset of 10% of each annotation sheet (usually several hundred samples) was cross-validated and Cohen’s Kappa coefficient was calculated subsequently. Cohen’s Kappa had to be equal or higher than 0.8 in order to obtain a satisfying result that could be later used for further analysis. This is according to the previously established methodology used also by Shing et al. (2018) [39], who showed that experts’ agreement in the task of annotation of suicidal content is around 0.8 of Kappa coefficient, while laypeople score only around 0.5 (see Section 2.3 for details).

When it comes to the agreement per each category, the categories with the strongest Kappa equal to 1.00 were typically Past Attempts, Traumatic Experiences, Cry for Help and Self Harm. In other categories, some significant changes in Cohen’s Kappa coefficient were observed after improving the instructions. Cohen’s Kappa for Complaint was initially varying between 0.42 and 0.79, and for Suicidal Thoughts it was between 0.59 and 0.68. After the instructions were improved Cohen’s Kappa for Complaint and Suicidal Thoughts increased to between 0.8 and 1. Throughout the entire annotation process the team participated in regular individual and group meetings where all the annotation discrepancies were pointed out and explained.

We also acknowledged and addressed the possible mental burden on our annotators. In particular, the annotators took part in workshops with a certified psychologist in order to prevent their mental discomfort.

### 3.4. Rhetorical Moves in Suicidal Context

#### 3.4.1. Theoretical Background for Rhetorical Moves

Most corpus analyses (see Section 2 for details) have been limited to quantitative descriptions of the data, which primarily focuses on describing the lexical and syntactic levels of the texts with the use of tools for morphosyntactic and lexical analysis. On the other hand, single-genre corpora make it possible to examine the entire corpus entries with the use of discourse analysis and rhetorical approaches. That does not necessarily preclude a more typical lexico-grammatical analysis but in accordance with the top-down approach, this lexico-grammatical analysis is preceded by a step in which communication goals are determined for encompassing text units, often encompassing text spans longer than sentences and corresponding to linguistically unified rhetorical moves. The corpus used in our project (see Section 3.2 for details) is comprised of posts from subreddits related to suicidal discussions (list of subreddits can be found in Appendix A at the end of the paper). These posts were not uniform in terms of genre, but they share common features as far as the subject matter and the form of a Reddit post is concerned. These individual texts are made up of complete, cohesive submissions written by a single person and can be approached from a discourse analysis perspective. Having established that, we have started the process of searching for suicidal declarations with the genre analysis approach suggested by Swales et al. (1990) [88] that was already used for describing suicide notes [72]. After that, we also applied standard qualitative methods, which in the case of research into suicide-related texts typically entail the use of LIWC. According to Swales’ theory, genre analysis can be seen as a three-stage process based on: identifying communication goals, identifying rhetorical moves that realise these goals and determining the rhetorical strategies that were used [88]. Reviewing the posts from the corpus from the point of view of communication goals shows that these texts are related to the subject matter of suicide, but they tend to be written with various goals in mind. Some contain requests for responses to particular questions (*How does everyone else deal with PMS?*; *Did any of you get prescribed any pills? Because mine don’t seem to be of much help*), others contain requests for: advice (*I don’t know what to do?*; *How can I break out of my prison?*), help (*Can someone please help me*) or attention (*I’m probably just on attention seeker but I don’t fucking care*). Among them, we can also find assertions informing about the author’s decision to commit suicide (*I wanna die now*; *I want to leave this place as soon as I can*; *I want to kill myslef and I’ve written my suicide note already*). In some cases, the communication goal is not clearly verbalised and can only be inferred from the complete utterance—a specific set of rhetorical moves. In spite of the fact that the communication goals across the collected texts differ, there are rhetorical moves that are common for many texts due to the standard post structure (e.g., a greeting, an introduction, thanking for reading the post) or due to the fact that they help realise a given goal, such as one of the two below:

- I want to ask for advice with mental health issues, therefore I describe my medical history (move: Providing explanation);
- I want to inform others about my decision to commit suicide, therefore I describe how my history of mental health issues has lead me to this decision (move: Providing explanation).

Big data analysis does not make it possible to holistically examine the posts in order to identify all of the existing rhetorical moves. Using this method with a high degree of specificity is not only time-consuming but also costly and makes it impossible to analyse the data in real time, which is particularly crucial when considering the possibility of conducting interventions for people at risk of committing suicide. The annotation procedure we established (see Section 3.3 for details) relates to the rhetorical move analysis methodology, while also considering the fact that the procedure needed to be simplified and adjusted to the analysed posts as they are consistent in terms of subject matter (suicide) but not in terms of genre (subreddits on various topics). The basis for the annotation process is the seven steps approach to discourse analysis [89] which was adjusted to the particular features of internet text analysis and validated with an initial test annotation. The final procedure entailed:

1. Identifying communication goals;
2. Segmentation (without paying particular attention to the boundaries as Internet posts tend to be unorganised);
3. Linguistic characterisation of the rhetorical moves identified in the segments;
4. Listing rhetorical moves that repeatedly appear in the analysed texts (while skipping the superordinate-subordinate hierarchy of rhetorical moves and steps);
5. Identifying elements obligatory for a suicidal declaration.

The annotation procedure therefore did not assume identifying all possible rhetorical moves in the analysed texts. Certain potential rhetorical moves may belong to genres that are of no interest to us. Even in the context of a suicidal declaration there might be moves that are not one of the obligatory, most crucial ones. This was confirmed through a stage of manual annotation, where a trained annotator tags the text on the basis of an initial assessment of the entire text, followed by assigning suitable rhetorical moves to the identified segments. Finally, we have identified the following rhetorical moves as obligatory and characteristic for suicidal declarations:

- **Suicidal thoughts:** information about thoughts of wanting to die or committing suicide;
- **Declaration:** information about the decision, or announcing of suicide;
- **Plan Method:** information about the planned suicide (time, plan, method).

#### 3.4.2. Examples of Rhetorical Moves in Context of Suicidal Declarations

In this section, we present examples of rhetorical moves representative for suicidal declarations annotated in the process of development of the SamuraiLabs RSQ Dataset. To avoid any personal information leak, the examples do not represent the exact samples from the dataset, but were anonymized and paraphrased to be representative and at the same time general enough to not be able of being searched online. Each separate example was taken from a longer context, and the specific word span for which each category was annotated was underlined.

##### Being Burden

**Example** **1.** *“Every day, I have numerous thoughts of simply disappearing or killing myself, as well as endless feelings of being a burden and disappointment."*

**Example** **2.** *“I’m just stuck in a self-destructive loop, and I’m a burden on your life and everyone else who has to put up with me every day, and one day you and my family won’t have to cope with such a wretched failure like me.”*

##### Complaint

**Example** **3.** *“I mess up anything I do, […] My mother is also there to remind me of how much of a letdown and embarrassment I am to her.”*

**Example** **4.** *“All in my life, including my whole life is only an act to keep others cheerful and I’m simply getting truly worn out on it. I feel also, that people watching me languish is a continuous torment for them as well, so in a way I’m finally giving them some closure as well”.*

##### Cry for Help

**Example** **5.** *“I don’t have any friends in my town, and I’m completely alone. I’m sick of feeling like this every day; it’s as if someone is ripping my heart out and I can’t stop it. Please, just give me someone to talk to."*

**Example** **6.** *“I am so lonely, I have no friends in my state, and I am ashamed to share what is happening."*

##### Declaration

**Example** **7.** *“I don’t have a future. I’m going to compose my suicide letter tomorrow and end it all at night.”*

##### Farewell

**Example** **8.** *“I just wanted to express that because the very last route I tried for relief was closed to me, I’m turning to my only remaining option. Death. I’m sure none of you are familiar with me. But, if anyone cares, I’d like to say farewell to everyone.”*

##### Past Attempts

**Example** **9.** *“I did and was additionally the casualty of some horrendous crap. I attempted to end it all twice but was saved each time.”*

**Example** **10.** *“Though I have tried to commit suicide in the past, I was never able to complete the act, which made me think I maybe did not really want to die.”*

##### Plan Method

**Example** **11.** *“I was thinking about what I should take in the event that I need to do. I have niquil, melatonin, lithium, gabapentin, and tylenol. what might work?”*

**Example** **12.** *“I’ve attempted slitting my wrists yet my knife didn’t dive adequately deep. I can’t hang myself, bc I don’t have a solid rope. If anybody has any ideas please im imploring you I need an exit from this shitty place please.”*

##### Self Harm

**Example** **13.** *“In this post, I will tell you about a new injury I have caused recently. I love getting hurt and I want to lose as much blood as possible (but not kill myself). I also don’t want many scars. Do you know what I can do?”*

**Example** **14.** *“After 3 months something of not cutting myself, my piece of shit self did it again. I just cut myself once on my thigh, I can’t believe I did it because of just one stupid grade.”*

##### Suicidal Thoughts

**Example** **15.** *“I don’t want to sit and wonder when I will hurt again. I want to die, but I won’t end my life.”*

**Example** **16.** *“Although I’m doing well these days, I still have a strong desire to end my life. It might seem scary, but it’s not. I’m doing a lot of things that I’ve wanted to do for a long time, and next week is my birthday. I can’t explain why, but something in my brain makes me feel as though I want to die. It’s weird and hard to explain.”*

##### Traumatic Experiences

**Example** **17.** *“First, let me briefly explain why I think suicide is my answer to life. My brother passed away when I was 13 years old. I watched him have an asthma attack and choked in front of my father and me. Neither the doctor nor the emergency room people were able to do anything and he died in the hospital. Later, my parents divorced and my mother left when I needed her most.”*

## 4. Statistical Analysis and Insights from Reddit Suicidal Query (RSQ) Dataset

### 4.1. Methodology and Analyzed Areas

After having the final version of the dataset collected, apart from using it to develop the rule-based suicidal text detection system, we also performed an in-depth analysis of the contents of the dataset. This was conducted to confront the dataset with the existing knowledge about suicidal language and analyse to what extent the contents of the collected data are in coherence with the existing knowledge described in previous research. Additionally, since our analysis was overseen by suicidal language experts, we hoped for making some new discoveries about such language, that would update the existing knowledge base and help in future research on this kind of language.

Although in the analysis we focused mainly on extracting as much of useful knowledge from the dataset itself, where it was necessary we also did additional analysis of Reddit data to check how our data extrapolates to a wider scale. Specifically, we analysed three aspects of the dataset.

Firstly, we checked the tendencies in the general activity of suicidal and non-suicidal users on Reddit. This could be useful for a variety of studies as well as in practice, as it reveals language-independent motion that drives each group of users.

Secondly, we used LIWC, or Linguistic Inquiry and Word Count, a tool for statistical analysis of corpora using a wide set of dictionaries. Using this tool has become a standard in psychological studies on language [90], and information it provides is also often used as features applied in machine learning algorithms [39]. We performed an in-depth analysis of correlations of words and categories which LIWC automatically annotates on text to find useful, meaningful correlations in vocabulary used in suicidal texts. However, we did not only look at the numerical data, but also actually looked at how the information annotated by LIWC corresponds to the actual meaning conveyed in such message. As LIWC in its text processing uses only simple lists of words, it could be possible that the words picked up by it are in fact used in completely different context.

Finally, we performed a standard topic modeling of the dataset to find whether there are differences in topics discussed by users of suicidal tendencies and others.

### 4.2. Tendencies Found in User Activity

To gain a deeper knowledge of the dataset as well as of the characteristics of suicidal users on Reddit, we analyzed the general tendencies in activity of suicidal users.

The first step consisted of an initial manual verification, finished on 2 July 2020. A subset of 185 users-authors of the samples considered as genuine suicidal messages (True Positives, or TPs, in contrast to messages that only seem like suicidal messages, but are not—False Positives, or FPs) in the dataset was selected and then checked whether the user was still active since posting the suicidal message captured in the corpus. Furthermore, if they were active, we also checked whether their most recent message was suicidal or not, and if they did not post anything since, we checked whether their account was abandoned (existing but without activity in the form of posting new messages), or whether the account was deleted. We did that based on the assumption that users who are not posting anymore or deleted their accounts might have committed suicide, while users who are still posting have not. Although we acknowledge that it is not possible to say for certain whether a user has committed suicide or not, by the rule of *reductio ad absurdum* (https://iep.utm.edu/reductio/, accessed on 2 November 2021) [91] we can say with certainty that they did not if they continue to post messages. This initial step focused on two combinations of speech acts, namely, Plan/Method and Farewell as well as Suicidal Thoughts and Cry for Help. The two speech act pairs were selected based on the hypothesis that the former pair should correspond to a high-risk group—someone who has made their plans and wants to say goodbye, while the latter belongs to a low-risk group—people that exhibit suicidal ideation, but wish to improve their mental health and are seeking help.

The hypothesis seemed to be confirmed by this verification as the Plan/Method and Farewell pair included markedly more users that were inactive and deleted accounts than the Suicidal Thoughts and Cry for Help pair as can be seen in Table 2.

Moreover, along with this step, we also checked how the speech acts co-occur with one another. One of the main takeaways of that was that the Farewell speech act does indeed co-occur more frequently with Plan/Method than Suicidal thoughts and vice versa for Cry for Help. These initial results suggested that repeating such an analysis for the entire corpus could prove fruitful, but it was also clear that such an analysis would have to be automated due to the size of the corpus.

The first automated analysis was performed on 14 July 2020. Although at that time the corpus was still not fully annotated, we decided to conduct the analysis as a proof of concept and whether the findings of the initial test still hold over a larger amount of data. This time we also looked at pairs of speech acts other than the two used for the initial manual verification, which could reveal other potential combinations that could be of interest in further analyses.

In order to facilitate the automated analysis, we gathered all the usernames of the users in the annotated part of the dataset as well as the users that posted the messages that were classified as FPs in the corpus and verified their activity using the Python Reddit API Wrapper (PRAW) (https://praw.readthedocs.io/, accessed on 2 November 2021). In the automatic classification of user activity, we checked (1) whether the user posted anything after the message matched in the corpus, (2) whether they did not and (3) whether they deleted their account. We decided to focus only on these categories after the manual verification as they seemed to provide the most crucial information regarding user activity. If the ID of the most recent message a user posted coincided with the ID of the message in the corpus, that meant that they did not post anything since and were deemed inactive. In the case the IDs did not match, that meant that the user posted new messages since. If accessing the user’s comment history was not possible, that meant that the account was deleted. There were also cases where the user’s accounts were suspended or otherwise inaccessible and as they only made up a small portion of the corpus, they were not included in the final analysis.

This step showed that the findings of the initial manual analysis still hold across a larger sample. The totals for each individual speech act (excluding all other speech acts except for Complaint as it appeared in the majority of the annotated messages and was deemed as rather insignificant) and combinations of critical (Plan/Method, Declaration and Suicidal Thoughts) and supporting (remaining) speech acts (with the exclusion of all other ones except for Complaint) were tallied. We also looked at combinations among critical speech acts, the occurrence of one critical speech act excluding only other critical speech acts (not excluding supporting ones) and the combination of at least one critical speech act with the supporting ones. Within each of these groupings we checked how many of the messages within either individual speech act or combination fall into one of the three categories—whether the user posted anything after the original message in the corpus, whether they did not or whether the account was deleted. Using these raw numbers, we prepared three sets of statistics—the percentage of the correlation between user activity and speech act (or their combinations) in relation to the total for a given speech act category, the percentage of the correlation in relation to the total for one of the three activity categories and the percentage of the correlation in relation to the total number of TPs in the corpus.

At the beginning of October 2020, once the entire corpus was annotated, we repeated the automatic analysis as described above on the entire corpus. Those results were then expanded halfway through November 2020 by using the data prepared for the purpose of estimating the age of the users in the dataset. Using this data, for each of the users that did post more messages after the one in our corpus we counted how many messages they posted. Then, in the tallies described above, in addition to the information on how many messages fall into the category where their author still posted messages afterwards, we established a more coarse-grained division into the groups of 1–5 messages, 6–10 messages, 11–15 and 16+ messages posted by the user since writing the one captured in the corpus. The groupings were selected arbitrarily, where 1–5 messages suggest that the user was largely inactive after posting the message in the corpus, while more than 15 messages suggest that the user remained active. The final results show a number of interesting tendencies, although they are not conclusive.

Table 3 and Table 4 show the distribution of the user status categories among the TP and FP parts of the corpus. Nearly 20% of the TPs in the corpus were written by users whose accounts remained inactive or were deleted since writing the message (7.24% + 12.42% in Table 3), while in FPs, the amount is closer to 15%. This behavior is then more common among TPs which means it could coincide with suicidal behavior.

Among the critical speech acts, we hypothesised that Plan/Method represents the highest risk group, Declaration represents the slightly lower risk one and Suicidal Thoughts correspond to the lowest risk group. Table 5 shows the distribution of these individual speech acts among the activity groups which supports this hypothesis. Account deletions and the lack of or decreased activity tend to coincide with the Plan/Method speech act much stronger than with Suicidal Thoughts.

The conclusions stemming from Table 6 are not as apparent as the ones discussed earlier, but generally support the assumption that across the combinations with different critical speech acts, the supporting speech acts of Being Burden and Farewell seem to correlate with a lack of or a drop in activity (more of the messages annotated with the combination of these supporting speech acts and a critical speech acts fall into the categories of ‘did not post after match’ or ‘1–5 Posts after match’). They also correlate with account deletions, although so does Cry for Help (which aside from account deletions scores fairly high in other categories corresponding to higher user activity). The speech act of Cry for Help and the category of Self-harm seem to be the opposite of the previous pair as they coincide more strongly with user activity (both in terms of the raw number of messages in the ‘Posted after’ category as well as in the number of posts that were sent since posting the suicidal message in the corpus). However, it is difficult to ascribe any significance to specific data points due to small data sizes (for example the combinations of Plan/Method with Self-harm or Cry for Help were very rare and so the distribution among the categories is not particularly representative of the properties of the combination) and as such, they are difficult to interpret and include in this analysis.

In order to obtain even more insight into the activity of users after they posted a suicidal message, we processed these messages that were posted after using the suicide declaration detection system described here.

To compare how these findings differ between TP and FP users, we counted the average number of all ‘posted after’ posts (across the total number of users that posted anything after the suicidal message within the TP and FP group respectively) and the average number of those ‘posted after’ posts that were classified by our system as suicidal. The results can be seen in Table 7. It is clear that while the average number of all messages posted after the suicidal one in our corpus in FPs is more than twice that of TPs, the situation is completely inverted when comparing the number of messages detected by our system as suicidal, as in this case, the average for TPs is more than twice that of FPs.

**Figure 2:**
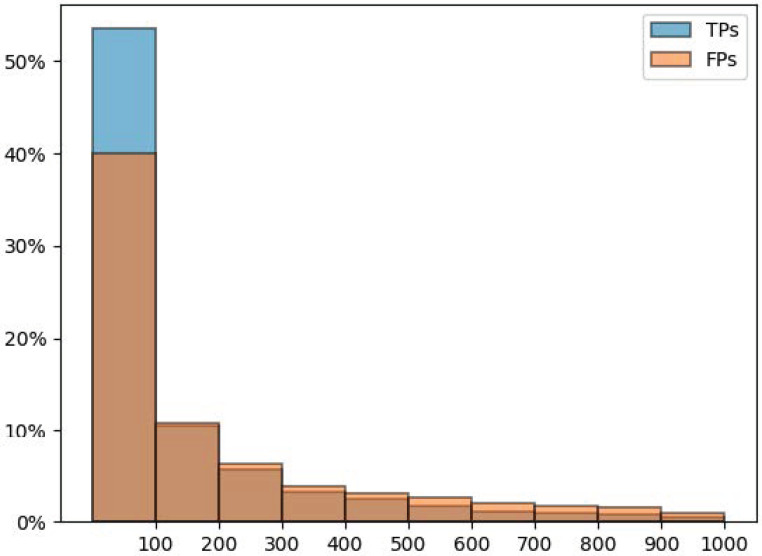
Percentage distribution of TP/FP users that posted within 1–1000 messages after the suicidal post.

To understand this data better, we also prepared a chart comparing the number of people within certain ranges of numbers of posts after the suicidal message, as can be seen in Figure 2.

While the chart in Figure 2 ends at 1000, in both groups there were users that posted even more than that. However, the number of users in ranges beyond 1000 was rather small and the trend seen here remained consistent even into these ranges, therefore we decided to exclude them in order to improve the readability of the chart. Users in the TP group vastly outnumbered the FP group in the lowest (1–100 posts after a match) activity range, trailing behind FP users in every subsequent range. This suggests that users that posted suicidal messages tend towards lower overall activity. A large number (around 40%) of users from the FP group is also in the lowest activity range, but overall they tend to appear more often in higher activity ranges than users from the TP group. The reason why the differences are so stark when comparing averages, but rather slim (apart from the lowest activity range) when looking at the chart might be partially due to statistical outliers. The most active user in the TP group posted 16,481 posts after the suicidal post, while the most active one in the FP group posted a total of 49,031 posts with a few other users posting more than 20,000 or 30,000 messages.

Overall, the data seem to provide support to our initial hypotheses. Moreover, it seems to indicate that the combinations of critical speech acts with the supporting speech acts of Being Burden and Farewell correspond to fairly high risk groups, due to the concerning drop in activity. The supporting speech acts express the emotions of feeling like one does not deserve to live or that others would be better off without them (Being Burden) and to the intentions of saying goodbye to the world (Farewell) and as such their presence is likely to indicate the fact that the author is close to committing suicide. Conversely, the speech act Cry for Help suggests that the author wishes to improve and is seeking ways of escaping their current situation. It also indicates that the user is willing to interact and to find support by working with other people. The Self-harm category is a different case—self-harming does not necessarily need to be indicative of suicidal intentions but instead, it can be its own pathological form of regulating stress or negative emotions [92,93,94].

### 4.3. Analysis with LIWC Categories

#### 4.3.1. LIWC—Short Description of Technology

LIWC (https://liwc.wpengine.com/, accessed on 2 November 2021), or Linguistic Inquiry and Word Count, is a text analysis software that provides simple statistics of the analysed texts. The statistics are provided for a large number of categories, beginning with simple ones such as word count, words per sentence, to more grammatical and syntactical, such as a number of pronouns, or negations, to more psychologically meaningful ones, such as words expressing or related to emotions (anger, anxiety), health-related (body, ingestion/eating, death), and many other categories [95,96], with overall 82 (eighty two) categories grouped in five larger category groups. All LIWC categories with their abbreviated labels used in our analysis was summarized in Appendix B at the end of this paper.

Despite an extensive number of dictionaries with which LIWC annotates the aforementioned categories on the input text, the processing method is fairly simple, namely, LIWC performs dictionary lookup on the input to decide whether an input contains a character string (word, etc.) from one of the dictionaries. This method is good enough in many situations but also introduces a number of potential disadvantages due to words being used in reality in different meanings, in metaphors, fixed phrases, jargon or slang expressions. We study these discrepancies in later sections. Despite such potential disadvantages, LIWC has been used extensively in a wide range of studies.

Some studies applying LIWC include psychology in general [95,97,98], studies on language of suicide victims in particular [36,37,38,82], using LIWC categories as additional features to improve machine learning algorithms [99], as well as multidisciplinary studies combining machine learning and psychology [39,40,100,101].

In our study, we primarily used LIWC to find meaningful correlations between categories found in different parts of analysed dataset.

#### 4.3.2. Methodology Applied in Analysis

In our in-depth analysis of the developed dataset, we applied LIWC categories to obtain several types of statistical information. In particular, we perform the following analyses.

##### Comparison of Raw and Relative Numbers of Each LIWC Category in Each Risk Group

Here, we compare simple straightforward scores of LIWC scores for each category in each risk group in our dataset. This is to find which category appears most often in each group in our dataset. Such information, despite being raw and thus difficult to confirm using statistical tests, provides a good insight into what to expect and whether the results are expectable (e.g., more topics related to death, health, or eating disorders in truly suicidal texts).

##### Normalized Correlations among LIWC Categories

There are two major problems with all previous work on applying LIWC categories in the analysis of suicidal texts. One is shallow analysis of LIWC categories. Second is uncritical application of those categories in the analysis.

Regarding the first problem, the researchers usually simply consider a category as specific to suicidal texts when it appears more often than other categories. However, LIWC analyses data only by doing a simple dictionary look-up/word matching and thus by definition is not capable of grasping context-based and metaphorical use of language. This means that LIWC scores might provide a wrong impression, which, together with the researcher’s confirmation bias can lead to wrong conclusions. To mitigate that, instead of only analysing the frequently appearing categories, we also calculate correlations among each category in each of the four risk groups (suicidal, high, medium, low). This allows us to analyse the dataset more deeply, since we can evaluate two categories at once and further confirm on specific examples whether the correlation is reliable, or whether it is a result of LIWC’s simplistic analysis—this partially solves the second problem.

Moreover, even if there is a correlation between two categories in suicidal texts, this still does not assure that such a category (or a pair of categories) is a reliable characteristic of the suicidal texts. We acknowledge that such a correlation could represent not the true property of the analysed language group (e.g., highly suicidal texts vs. moderately suicidal texts), but could be a more general property of the whole dataset, or of the group itself (e.g., users in gaming community talking about dying in a game). Therefore, we additionally normalize the correlations by taking the difference of correlation scores calculated separately for True Positives (TP, actual suicidal texts) and False Positives (FP, texts that look as suicidal at first glance but are not based on their wider context). This normalization was conducted to assure that, even if a pair of categories correlate with one another, this correlation is specifically typical only for either TP or FP. Next, we look more specifically at those correlations which were specific to only TP or FP (the absolute difference of correlations TP vs. FP was high) to discuss their potential meaning, and contrast them with existing knowledge based on suicidal language. Although we understand that correlations do not represent causal relations, this way of normalization assures a much higher chance for the correlation to reflect real world relations. As a final step in the analysis, we look at the specific examples that were representative of such normalized correlations. We do that to both visualise how the correlation is represented in real language, as well as to estimate to what extent LIWC is capable of grasping the true use of language, and is usable in studies on suicidal text analysis. The specific step-by-step procedure for the elimination of irrelevant categories was outlined in Algorithm 1. The threshold set in the method (α=μ+σ×5) was set experimentally and can be raised (×6, 7, …) or decreased (×4, 3, …) depending on a specific dataset, and the needs of the researchers (more coverage for analysis, or more strict conditions), as well as the difficulty of a task, and compared categories.
**Algorithm 1** Procedure for specifying relevant categories1:**for** correlation_type **in** [Spearman, Pearson, Kendall] **do**2:    **for** risk_group **in** [suicidal, high, medium, low] **do**3:        **for** each LIWC_category *c* (see Appendix B) **do**4:           **for** suicidal_or_not **in** [TP, FP] **do**5:               Calculate correlation ρ between all other *c*6:           **end for**7:        **end for**8:        Calculate $|\rho \_c_{TP}-\rho \_c_{FP}|$ for all *c*9:        Calculate average_of_correlations μ10:       Calculate standard_deviation_of_correlations σ11:       Calculate threshold α=μ+σ×512:       For final analysis choose LIWC_category correlation pair ≥α13:   **end for**14:**end for**

##### Applied Correlation Measures

To calculate correlations, we applied all typically used correlation coefficients, namely, Pearson’s product-moment correlation coefficient [102], Spearman’s rank correlation coefficient [103] and Kendall’s rank correlation coefficient [104] (later called Pearson, Spearman and Kendall, respectively). However, as results for distributions of scores for Kendall were identical with Pearson, in analysis we compared only Pearson and Spearman, however all scores including Kendall were also added as additional data attached to paper. Difference between Pearson and Spearman is significant in the sense that Spearman represents only a correlation of ranks, thus not taking into account actual score distributions. This can sometimes be more useful as variations in sizes, numbers of sentences and sentence length (words per sentence) among the compared dataset sub-groups can differ thus “diluting” the Pearson correlation due to such dataset discrepancies. In such situations Spearman’s results are more reliable. All correlations were also supported by statistical significance analysis. The notation of statistical significance is represented next to the correlation results as: * *p* ≤ 0.05, ** *p* ≤ 0.01, *** *p* ≤ 0.001, **** *p* ≤ 0.0001.

#### 4.3.3. Discussion on Results Obtained in Statistical Analysis

##### Analysis of Normalized Correlations among LIWC Categories

In this section, we present the insights we had by analyzing the dataset according to the methodology of Normalized correlations among LIWC categories described in Section 4.3.2. We analysed each risk group separately to either confirm existing knowledge or find new, or even unexpected knowledge in texts containing suicidal declarations. Each potential finding was checked by looking at a number of specific examples from the dataset to either confirm or reject the hypothesis.

##### Suicidal

One of the categories for which the difference was the highest, both for Pearson and Spearman correlation in the group of highly suicidal subreddits (Table 8), were Positive emotions (Love, nice, sweet) and Reward-related expressions (take, prize, benefit). Although at first glance this might seem contradictory, a greater frequency of positive emotions is in fact a well-known characteristic of farewell letters. These often represent gratitude towards family members and friends, who have supported the individual for a long time. Moreover, texts written by individuals who made the final decision, often contain phrases related to achieving the goal, which often is represented with reward-related phrases. The difference is also visible in both Pearson and Spearman, yet weaker in the latter.

Some of the examples include: “Finally got an opportunity to do it. Gonna throw myself off a building on [DATE]. Thank you guys. Your’e the only reason I made it this long”, “I put my shotgun to my chin and pulled the trigger (not bullet in the chamber) and it felt good”, or “i’m going to kill myself before i turn 20. it’s the best option for me. end it early while i still can”.

On the other hand, there were also cases where the correlation appeared by mistake. For example, in the sentence “Corona take me away. I no longer want to be alive”, the word ‘corona’ (crown) was considered by LIWC as a reward. In addition, in question such as “Is this enough to successfully kill myself?” the word ‘successfully’ was triggering both positive emotions and rewards, which, looking at the context, is not an appropriate interpretation.

The use of emoji (graphical emoticons) together with Netspeak (internet slang) suggests the user’s lighthearted attitude towards the written content, thus it can be considered as a valuable negative predictor (Table 9).

Similarly to emojis with internet slang, the use of emoji together with fillers (aaa, uhmm, etc.) suggests a lack of decisiveness and in general a lighthearted attitude (Table 10). Thus, despite no correlation in TP, it had a sufficiently strong correlation in FP to consider it as a valuable negative predictor.

A good example of the fact that one category alone does not have a predicting capability is the correlation between Internet slang and motion-related expressions (arrive, car, go) (Table 11). Despite the occurrence of the popularly accepted Netspeak, in suicidal texts, the slang is usually of much lesser intensity, yet with the use of motion-related expressions, it shows a sufficiently strong correlation in TP. Here, typical examples of Netspeak include such contractions as ‘going to’ → ‘gonna’, or ‘I dont know’ → ‘idk’, etc., while movement and motion-related expressions include ‘go [kill oneself]’, or ‘stop [living]’, as in the following sentences: “I want to die. I hate my life. And I think imma set a date and go through with it”, “im gonna hurt myself. i dont want to but i cant stop myself”, “[…] idk what im going to do next. If I end it this is my goodbye and I’m sorry”, or “I get off of work in three hours. When I get home I think I’m gonna dip. […] idk how to start saying goodbye to people without drawing suspicion”.

In suicidal messages colons (:) often represent time, either reported, or planned for suicide (“It’s 3:24 in the morning here in Portugal, and im having suicidal thoughts!”), or when used for further explanations (“[…] in a few words: I’m afraid of food. I just want to die”) (Table 12). They are also often used in emoticons (“:(”). Together with exclamations (!) they usually express frustration, often because of someone’s bad advice (“They always say the same thing: life is wonderful, […] good sides make it worthwhile, etc… The thing is- I already know that! […] I’m just done with living”, or “here’s the bad thing: […] I have times where I just…just get pushed off the edge!”). On the other hand, suicidal Reddit posts can sometimes be very long (several thousand words), with whole life stories explained leading to the suicidal decision, and thus sometimes the two categories simply appear together without any specific direct relation. This shows another disadvantage of the LIWC, namely, the lack of structured analysis incorporating the inter-relations between categories.

Words representing Social processes (mate, talk, etc.) correlated negatively with Authenticity for both Pearson and Spearman, more strongly in FP, than in TP (Table 13). *Authenticity* is defined in LIWC in the following way: “When people reveal themselves in an authentic or honest way, they are more personal, humble, and vulnerable. The algorithm for Authenticity was derived from a series of studies where people were induced to be honest or deceptive [105] as well as a summary of deception studies published in the years afterwards [106]”.

Since the correlation was negative, it can be hypothesized that the more the users write about their social life, the less authentic they seem, and vice versa. Although this assumption seems reasonable, the tendency of the correlation was the same in both FP and TP. It is also a reasonable result when we consider that basting about one’s personal life in non-suicidal context has a different quality than when a suicidal individual explains their life story leading him to the tragic decision.

However, although the tendency of the correlation for this pair was consistent, and examples are confirming the hypothesis, LIWC scores for those categories for specific Reddit posts were low compared to other category pairs. This might be due to the difference in calculating each of the two categories—Social processes are simply calculated on the basis of the number of social words in the sentence, while Authenticity is calculated using a specific algorithm.

Exclamations correlated positively with Negative emotions (Table 14), and more precisely, with Anger-related expressions for FP rather than TP. Firstly, it is reasonable, if not obvious, that anger is often expressed on the internet with the use of such linguistic tools as exclamation marks, therefore, the fact that there is some correlation is not surprising. It is also predictable that such correlation will be more expressed in FP, rather than in TP, since truly suicidal individuals are less likely to scream their negative emotions out (and thus reach a relief), but rather would bottle them up. In this sense, the correlation is in line with present studies on writings of suicidal and depressive individuals. However, since the correlation is not strong even in FP, it cannot be considered as a strong negative predictor, but rather a supporting one.

Death-related expressions and 1st person singular (‘I’) is the most obvious correlation to expect in suicidal and suicidal-looking texts (“I want to kill myself”, “I want to die”, etc.) (Table 15). LIWC also properly shows that this feature is more pronounced in truly suicidal messages. However, due to the prevalence of this characteristic, it should be taken with caution, as it also occurs in plentiful numbers in pseudo-suicidal texts, such as on gaming forums (“I just died”).

##### High

One of the most conspicuous differences in High-risk group was between categories Death (words related to death: bury, coffin, kill, etc.) and Focusfuture (words related to the future: may, will, soon) (Table 16). Since correlation in TP for this pair was sufficiently and significantly strong (0.42722 ****), while for FP there was no correlation (0.03902, although not statistically significant), we can consider this correlation pair as meaningful. Often in suicidal posts, users write phrases like “I will soon killl myself”, “soon my life will end”, or “I may kill myself tomorrow”, etc.

For Spearman, the difference was not observable, which means that the strength of the correlation is not due to the order (ranking) but the actual distribution.

Another strong candidate was the pair of Colon (:) and Dash (-, –, ―) in High-risk category (Table 17). The correlation was especially significantly strong in TP (0.50178 ****), and significantly weak in FP (0.11621 ***), which suggested a meaningful correlation in suicidal texts. However, as this correlation was between two sets of punctuation marks, we did not have high hopes for this pair result.

In practice, users were, e.g., using sad emoticons (“ :-( ”, “ :( ”), which contain colons and often dashes as well, to express their sad emotions. Moreover, there was an unrelated, yet correlated, usage of colons and dashes, used in explanations of users’ mental states, or specifying time periods in personal stories (“For 3–4 days a month, I’m a mess”.)

However. Spearman’s correlation was similar for both TP and FP, which could suggest that the differences in data distribution and differences among other LIWC features caused the high difference between Pearson’s correlations. Therefore, although this category pair seems meaningful, further experiments are needed to confirm whether this result was not purely data-dependent.

Next, potentially viable pair of categories was Apostrophes (“, ”) and Death (Table 18). We noticed that as the texts in the dataset often contain words related to death, and thus the LIWC category of Death often appears in correlations. This means, that although this category could provide plentiful new knowledge, it could be equally misleading.

However, this time the stronger correlation was in FP (0.35013 ****) rather than in TP (−0.00992), which would rather suggest a meaningful non-suicidal correlation. This could be useful in filtering out messages that despite showing high occurrence of death-related topics are in fact not suicidal.

A stronger correlation for words related to death and apostrophes together in FP could suggest that people sometimes use those words in quotation marks thus indicating not being serious while using it. It could also be used to quote famous death-related quotations from literature or poems. Some people might use apostrophes for citing specific ways to die.

However, the most frequently seen explanation of this correlation was in the use of death-related words with contextual use of personal pronouns and grammatical contractions (I’m, he’s, he’d, don’t, etc.), such as in the following examples: “I’m dead”, “I really wish he’d die from covid-19 or anything else”, “It’s quite the hell”, “I hope I don’t die from stress”.

However, since there was no sensible correlation found for this category pair for Spearman, it should be checked in the future whether this correlation was not data-dependent.

A weaker, but meaningful correlation was noticed between categories Periods (“.”, “…”, etc.) and Death ( bury, coffin, kill, etc.) (Table 19). The correlation was moderate in TP for Pearson (0.27252 ****) and weaker for Spearman (0.11526 **), while there was no correlation for FP.

Since higher correlation was found in TP. Specific examples showed that when people write about death they use an ellipsis (…), such as “And then everything will come to an end …”, or “I’m suicidal and this is my last bit of purpose and hope …”. However, in large part death-related words in TP were simply used in carefully written full sentences. Although a far-fetched explanation could suggest that users who are suicidal tend to use the full stop (period) character to symbolize their suicidal decision, a weak correlation for Spearman suggests that the correlation rather might be data-dependent, and thus analysis on a different set of data would be desirable.

Another valuable correlation was between the categories Ingest (Biological processes → Ingestion, containing ingestion-related words, like: hungry, hungrier, hungriest, dish, eat, pizza) and Leisure (Personal Concerns → Leisure, containing words related to leisure activities, cook, chat, movie) (Table 20).

Ingest and Leisure correlated significantly strongly with TP, suggesting people write more about eating and leisure activities in suicidal messages.

Eating disorders have been correlated with depression and suicide. In fact, there was a number of examples proving this, such as in: “I’ve been dieting to help myself feel better about myself, but my mom is catching on, so she’ll probably make me eat and gain weight”. or “I have to be at work in an hour and I cant even bring myself to get ready or at least eat something”.

However, in most examples this suggest a misunderstanding because of words such as “talk” (leisure) and “drink”, such as in e.g., “I dont have anyine to talk to, so I will drink myself to death/swallow a bunch of pills and die.” Although in principle this confirms the correlation, considering “lack of having someone to talk” as Leisure and “drinking”, in the sense of being drunk with alcohol, as Ingestion, seems more likely to be caused by a lack of contextual processing in the LIWC software.

This is confirmed in Spearman, where for TP and FP the two categories were similarly correlated (TP = 0.35925 ****, FP = 0.33136 ****). Spearman also shows moderately strong correlations, which suggests that the distribution (many highly scoring samples) influences the results.

A strong candidate for a valuable correlation was between Death (words related to death, such as: bury, coffin, kill, etc.), and Informal language (Table 21). Additionally, the Informal language category is a super category for several sub-categories such as Swear words (fuck, damn, shit, etc.), Netspeak (btw, lol, thx), Assent (agree, OK, yes), Nonfluencies (er, hm, umm), and Fillers (I mean, you know). Therefore, we checked additionally whether one of these categories is more influential than others.

In general, the correlation was significantly strong for TP (0.44829 ****), rather than significantly weak in FP (0.11313 ***), which suggests that more informal language is used with death-related words in actual suicidal texts. This is a good candidate for a strong predictor.

However, a closer look at which of the Informal language subcategories revealed that there was no visible pattern of correlation for any of the separate sub-categories. Only when all of them are combined a clear correlation was visible. Thus the super category as a whole is a stronger predictor than any of the sub-categories. This is an interesting discovery in the sense that a too fine-grained analysis might sometimes blur the image, while a more coarse-grained look at the data might reveal some interesting findings. Unfortunately, there was no meaningful correlation for Spearman, thus there is no confirmation for this correlation in rank-based correlation. Typical examples for this correlation were, e.g., “I just want to fucking kill myself” (Death with Swear words), or “I wanna die now” (Deat with Netspeak).

An interesting correlation was observed between Death and Discrepancy (should, would, etc.). There was a strong positive correlation in TP (0.51509 ****), and weak in FP (0.22868 ****) (Table 22). This was clearly visible in suicidal messages (TP), where users often used phrases like “I should kill myself”, or on the other hand, ask questions like “Should I kill myself?” Thus, the correlation could be stronger there. In FP users also use such phrases (thus the correlation exists), but in a metaphorical sense, and the co-occurrences are not that frequent (thus the correlation is lower in FP). Spearman’s correlation did not show a strong correlation, but the tendency was similar (TP > FP).

Death also strongly correlated negatively with Word count for Spearman (Table 23). This would suggest that when sending actual suicidal messages and talking about death users tend to write shorter sentences. Pearson revealed a similar yet weaker correlation, however, since the correlation is stable, we can say that this could be a strong predictor. Shorter sentences could be more indicative of a suicidal attempt.

On the other hand, Colons (:) strongly correlated with Exclamation marks (!) in FP for Spearman (Table 24). Since the correlation is stronger for non-suicidal messages, this information should be treated rather as a disambiguator than a predictor.

Since it is easy to recognize each category in the text (colon, excl. mark), the information could help detect FPs. Unfortunately, the strength of the correlation is not high enough to simply use it as a rule for detecting FP. However, it could be useful to check if the presence/absence of those two features together changes the results in machine learning approaches to suicidal text detection. Pearson showed a similar, although weaker tendency in correlation.

A moderately strong negative correlation was observed for categories Death with 1st person plural, or “we” in short, which contains such words as “we”, “us”, “our”, “ours”, and is a subcategory of Personal pronouns (Function → Words → Total pronouns → Personal pronouns → 1st person plural) (Table 25).

Both correlations are negative, but the only one considerably meaningful is for TPs. This means that the less 1st person plural pronouns are there and the more death-related words are in the message, the more it is probable for it to be actually suicidal.

From the psychological point of view, “we” is a word building community, so also strength. People who want to commit suicide, usually feel alienated, alone, lonely, thus would use such words less often. This tendency is clearly visible in examples, although it must be noted that 1st person plural pronouns do appear in suicidal messages from time to time.

Similarly to the correlation between Deat and We, there was a weak, yet potentially meaningful correlation between Death and 3rd person singular pronouns, or in short “Shehe”, which is also a subcategory of Pronouns (Function Words → Total pronouns → Personal pronouns → 3rd person singular), and contains such words as “she”, “he”, “their”, etc. (Table 26).

TPs moderately characterize themselves with a higher frequency of death words and at the same time lower frequency of she/he pronouns. An explanation for this could be that users who want to commit suicide would usually talk about themselves, thus would use she/he pronouns less frequently. However, even in our data, there were many people who wrote they want to commit suicide because of someone else (family member, harassers, etc.). These people use such she/he pronouns more often.

##### Medium

High positive correlation for FP suggests that users express their feelings in a variety of ways, including rhetorical use of suicidal phrases like “I want to die” in a non-suicidal context, often connected to health-related complaints (Table 27).

Messages typically included phrases like “I hate my life”, with “Bio” mainly referring to the ‘life’ keyword, which was not particularly representative. Other examples included, e.g.: “I really need help, I feel so alone so sad. Don’t wanna be alive no more. Need drugs to live because nobody can love me. This hurts”, or “hope that i get coronavirus and die from it because of how shitty my life is going lately”.

Anger, which is related to affect, showed a strong positive correlation with Biological processes in non-suicidal messages (FP) (Table 28). In rhetorical FPs, users expressed their anger (also related to health and/or their looks) by saying they want to die—e.g., “The pain is so big that I feel I want to die” etc., which should not be treated literally. In TPs, they were expressing angry while adding they wanted to die in a literal sense.

The majority of the examples included the ‘life’ keyword as representative of the Bio category. Some others included ‘face’, which is more representative, or ‘sick’ in the sense of ‘being sick of something’, also ‘to face something’ as a verb etc, ‘guts’ as in “I don’t have guts to do it”. Crucially, ‘bio’ often did not refer to a specific biological state (‘life/death’ are too general), but was annotated on words used in metaphorical meaning.

Some examples from FP include: “I fucking hate my life”, “Every girl that sees my face has no interest. Why shouldnt I kill myself?”, “I have no idea why I’m anxious, it’s a week now. I’ve not been to uni[versity] either. I have no idea what the hell is wrong with my brain. I couldn’t sleep much, now I can’t get up from bed. I fucking hate it”.

While actual suicidal messages (TP) included: “Time and time again each day I am reminded of how much of an ugly loser I am. I have no real life friends. I do not deserve to be happy or have a good life. I deserve to kill myself and suffer”, “I’ve been watching Unbelievable on Netflix and I just have so many traumatic memories of my assault/rape flooding back. I just want to die. I’m feeling impulsive today and I’m afraid I might do something rash. […]”

Similarly to the correlation Anger with Bio, Anger also correlated with the Health category in FP (Table 29).

This is due to the fact that in FP, users express their anger (often related to health issues) by stating rhetorically that they want to die, e.g., “The pain is so big that I feel I want to die” etc., which should not be treated literally. An additional example for this would be: “I don’t want to kill myself but sometimes (every night) the pain gets so bad that I cry myself to sleep again. I don’t want to die but I am going to end up killing myself because the pain is too much”. Additionally, in some contexts users expressed their wishes to die of some sickness (often coronavirus), such as in: “I honestly hope that i get coronavirus and die from it because of how shitty my life is”.

High positive correlation between Anger related expressions and 1st person pronouns in FPs suggests that users either express their own anger towards some external object (“I hate …”.) or express anger towards themselves (Table 30). Typical examples from FP confirm that, as in: “I hate my fucking life”, or “I fucking hate dating I’m going to die alone”.

As this correlation is more represented in FPs, this pair of categories could be a useful disambiguator for messages in Medium risk group.

Biological processes (eat, blood, pain) and Negative emotions (hurt, ugly, nasty) correlate positively in FP more than in TP (Table 31). In FPs, users often express their negative emotions related to health issues by saying metaphorically that they want to die, e.g., “The pain is so big that I feel I want to die” etc. Like in many cases for correlations with the Bio category, the meaning of biological processes is broad and includes ‘life’ as well as metaphorical references to body parts. All FP cases of “I want to die” represent this category as well.

Examples from TP include: “I hate my fucking life”, “Even with medication i’m still convinced that everyone hates me […] I hate my life why this disease had to choose me?”. While examples from FP, where the correlation is much stronger, include, e.g.: “I want to die I can still pass the course but this is so fucking stupid oh my god im so sad :/”, or “My life is just shit. I am terrible at school, my parents always yell at me, I am sad all the goddamn time for no reason at all. All I want to to is bake bread and die”.

The correlation for Death and Discrepancy (should, would—function words related to planning) is sufficiently strong for TP for both Pearson and Spearman (Table 32).

Although Pearson correlation for FP is also strong, it is negligible in Spearman, thus we can say that more meaningful is the Pearson correlation for TP, which can be considered as a strong predictor. “I should just die”, “I just want to die”, or “I’m planning on killing myself tonight. I keep fucking things up for everyone. And I’m a freakshow. It would be better if I was dead”. FPs include either rhetorical use of ‘I wanna die’ cases, or some speculations that one could die unintentionally, such as in: “Plus I’m stuck at work with comprised health. I could be dead soon”.

1st person pronoun (I, me, mine) moderately correlates with Sexual words (horny, love, incest) for FP (Table 33). In general sexuality is more common in FPs, which would suggests that users talk about their own sexuality and sex-related problems and desires which may coincide with rhetorical figures of wanting to die. However, few examples are actually sexual—it is usually about the use of word ‘fuck’ being used as an expletive.

A similar situation to the correlation between Sexual words and 1st person pronoun was between Sexual words and Personal pronouns (I, them, her), which is a super-category for 1st person pronoun, and influenced the result in this case, thus needs not be considered separately (Table 34).

##### Low

Comparing to previous risk group categories, messages in Low are mostly non-suicidal, and those which appear in this group can be expected to have different characteristics than those messages which appear on Reddit channels typically used to post suicidal posts. Therefore, we expected vividly different LIWC categories to stand out.

A category pair for which a vivid correlation was found was the pair of Health (containing words such as clinic, flu, pill), which is a subcategory of Biological processes concerning health and illness, and Money (containing words such as audit, cash, owe), which is a subcategory of Personal Concerns dealing with financial matters and the worries that come with them (Table 35).

There was a strong positive correlation in TPs with a large difference between TPs and FPs, and no correlation in FPs. It would seem that in the TPs from the Low category people tend to discuss matters related to financial or health issues or both (large costs of treatment leading to a difficult financial situation). These descriptions might function as explanations for their suicidal state.

Some of the examples contained people mentioning their struggles with health or financial matters. There were also samples which combined the two issues: “I can’t afford to go to the doctor” or “My parents obligated me to go this september we do not even have the money and I am failing most of my exams and I just want to stop living”. Mentioning these difficulties serves to contextualize the authors’ suicidal tendencies and provides an explanation for their mental state. On the other hand, this pair also includes certain messages that do not correspond to discussions of money or health-related issues such as “Life isnt worth living”, in which the Money category is realized in a metaphorical meaning.

A correlation similar to the above Health and Money was the pair of Money and Bio (which includes words such as eat, blood, or pain) and represents Biological processes, which is a larger category concerning health, body, sexual and eating matters (Table 36).

Pearson correlation for this pair in TP was significantly moderately strong and the difference was large, yet with no correlation in FP This pair and its correlations are rather similar to the previous one, but rather than just health issues the discussions here are somewhat broader, and revolve around sexual issues or eating disorders.

Due to the similarity between this pair and the one preceding it, there is a lot of overlap between the two. The content of the messages correlated with this category pair is somewhat broader compared to the previous one, with the first example listing the ways in which the author prepared their body as part of their suicide plan. The same issue with the metaphorical use of money-related phrases as in the previous category pair was also noticeable here, as well as the phrase such as “Life isnt worth living”, which also correlates with it.

Another standing out pair were the categories of Informal language (a general super-category spanning different subcategories of informal language such as netspeak, nonfluencies, etc.) and Fillers (I mean, you know), which is one of the subcategories of informal language (Table 37).

Strong positive Pearson correlation in TPs, weak positive correlation in FPs, large difference (with smaller difference yet similar tendency for Spearman), point out to simply a correlation between a super-category and subcategory. It correlates more strongly with TPs, which could suggest that people are more informal TPs, perhaps as a coping strategy. Or since they’re writing on subreddits in the Low risk group category there’s a need to tone down the serious content of their posts, such as in the example such as “anyways i wanna die”.

Many of the messages in this correlation pair are quite short and indeed use informal language or fillers, but their content does not seem to support the above hypothesis. They are rather straightforward suicidal messages that are simply written in a fairly informal way, nothing about them suggests that the authors are trying to tone down the serious content of these messages. At the same time, many of the messages correlated with this pair are also quite long and they do not read as particularly informal, exhibiting proper use of punctuation, standard vocabulary and full sentences, they just use a fair amount of contractions but even there it is not an unusually high amount.

Second pair related to Filler and Informal language in general was a pair of Filler and more specific from the Informal language category, namely, Non-fluency (er, hm, umm), which is one of the subcategories of Informal language (Table 38). There was a strong positive Pearson correlation in TPs, and no correlation FPs, with a large difference.

The hypothesis in this pair is nearly identical to the previous one and so are the conclusions with the caveat that in general the messages in this pair appear to be longer on average than in the previous one. At the same time, apart from the use of certain nonfluencies or informal language in general, they do not stand out in any particular way in terms of the overall content of the messages.

Another pair for which there was a larger difference in correlations between TP and FP was Body (cheek, hands, spit, etc.) which is a subcategory of Biological processes concerning specifically the body, and body parts, and Power (superior, bully, etc.), which is a subcategory of Drives, containing words dealing with power dynamics (Table 39).

Positive, yet not overly strong correlation in TPs and no correlation in FPs suggests appearance of discussions of ones’ body and issues related with it as well as potential imbalances of power between interlocutors or characters in a story described in a Reddit message.

Although many messages adhere to the above explanation, unfortunately, a closer look at many other messages revealed that especially the way the power-related words show up in these messages does not have a lot to do with actual power imbalances between people but it is rather used by the authors to discuss their own powerlessness, as in “I’m ugly unlovable and apparently unloving and unbearably annoying no one would want me and I must die soon cuz I can’t be alone anymore I just can’t someone help me kms”. One other way in which body-related words appear in these messages is the discussion of the specifics of suicide plans, as in: “So I guess I’ll just jump, and then snap my neck”.

Common Adjectives (free, happy, long) also correlate with Swearing (fuck, damn, shit—subcategory of informal language) more for TP than FP (Table 40).

3. Correlation strength and Hypothesis Specifically, there was a moderately strong positive correlation in TPs, and weak positive correlation in FPs. This suggests that the posts in TPs might be more descriptive with a higher frequency of swear words, which could signify frustration and anger.

This pair shows a straightforward correlation with the adjectives present in these messages, describing how the authors feel about themselves, their mental state or their outlook on the world and life. The swear words on the other hand also function as adjectives (as in: “The world is so fucked up”) or serve to strengthen the overall message and express anger, frustration or helplessness, as in: “I want fucking die”, “i wanna kill myself how’d i let myself be sick goddamit”, or “I’m fucking tired of living …”.

Adjectives correlate with Sexual words (horny, love, incest—subcategory of Biological processes, terms concerning sex and sex-related matters) similarly as with Swearing (Table 41). This strong positive correlation in TPs, weak positive correlation in FPs suggests that the posts in TPs might be more descriptive with a higher frequency of sex-related words, which could mean long and specific descriptions of sex-related issues are more common in TPs.

Descriptions of sex-related issues can also be found among the messages representative for this pair. Usually, they serve as an explanation for the author’s suicidal tendencies, as in: “I don’t think I’m a good person. I was raped when I was six years old and raped again on my 21st birthday”. Sexual words also function as a form of verbal self-harm, as in: “I’m not even strong enough to commit suicide I’m a fucking failed abortion But tonight Im actually doing goodbye world”.

Unfortunately, the messages representative for this pair also reveal a weakness of the LIWC tool—there is a major overlap between the messages in this pair and the adj_swear pair, in cases where “fuck” or “fucking”, used simply as swear words, are also interpreted as sexual words. This can be seen quite clearly in the following example: “fucking fuck why am i sad at home this fucking early shit fucking fuck. i dont know why im sad. i just … am. nothing bad’s happened. nothing big happened, so why the FUCK am I sad. FUCK I HATE LIFE”.

Interestingly, Anger related words (hate, kill, annoyed), which is a subcategory of Psychological processes, strongly correlate positively in Low risk group with Biological processes (eat, blood, pain, etc.), which is a super-category concerning health, body, sexual and eating matters (Table 42).

Strong positive correlation in FPs, and weak positive correlation in TPs suggests a combination of words that can both describe a negative physical state as well as violent video games, e.g., in the community of gamers.

This category pair is more common in FP posts, however it does not only correspond to gaming-related messages. Major part of the FP messages seen for this pair has their authors simply vent their frustrations and anger about the world, particularly in sentences starting with “I hate …”. The TP messages showing for this pair are somewhat similar to the ones found in the adj_swear and adj_sexual pairs, with swear words signifying anger. Some of the typical examples are: “I’m either killing myself or someones else. I don’t fucking care about anything anymore, not a single fucking thing and I’m tired of this shit I’m fucking ending it”. On the other hand, FP messages (similar in wording to suicidal, but not actually suicidal) contain such messages as: “I hate my fuckin life lol”, “fuckin hate living in this house”, or “I’m fucking dying lmao” (humorous or sarcastic).

Last pair of categories for which there was a sufficiently strong correlation in TP and a non-negligible difference between TP and FP was for Home (kitchen, landlord), which is a subcategory of Personal concerns, and concerns matters related to home and maintaining it, and Leisure (cook, chat, movie), another subcategory from Personal concerns, containing words related to relaxation (Table 43).

Strong positive Spearman correlation and a weak positive Pearson correlation in TPs, with high difference with FPs, could suggest that these terms are brought up in TPs more often either due to issues with the authors’ home situation or because of an inability to relax. It could also signify that the authors have certain issues in these areas or find that the situation at home does not support them or help them with their mental issues.

The particular examples show that this category pair does correspond primarily to home-related topics, usually in the form of concern or issues with the authors’ family. Users write about their desire for normal family life or friendship as well as their desire not to cause the family any problems or concern, such as in the following example: “I don’t want to live anymore and if that takes me then my family may see me as less of s failure”. If leisure is clearly mentioned, it is typically in the context of it being unattainable, due to circumstances having to do with home, family or other people, as in the following example: “But my misophonia sucks. I don’t have my room, live in small house and everyone has to always shitting eat or drink something or somwthing that triggers my misophonia and everyone except my brother is a trigger… […]”

##### All

We also analyzed the correlations for all texts, without dividing them into risk categories. This was conducted with two goals in mind. Primarily, we wanted to see what are the novel and overall most general correlations, not specific to risk categories. Secondly, we wanted to confirm the strongest and potentially the most informative correlations from the category-based analysis. Specifically, those correlations which reappear also in this overall analysis would be those to which suicide counselors and practitioners should put the most attention when making an assessment of a suicidal message.

The division into four risk categories (Suicidal, High, Medium, Low) was also an important step from the point of view of performing *information triage* [107] for future use by suicide experts and counselors. Information triage is a recently developed approach to the processing of information on social media with the goal to efficiently provide useful information to experts for more detailed analysis. For example, Ptaszynski et al. [107] performed information triage on Twitter during natural disasters (earthquakes, floods, etc.) to extract only tweets that were sent from actual victims of the disasters during the event with the goal to provide this relevant information directly to rescue teams.

In the context of suicidal messages, the division into suicide categories provides additional information to suicide experts and counselors, which can help disambiguate potential pseudo-suicidal messages classified initially as potentially true suicidal messages. However, having the correlations analyzed from the overall perspective as well is also important. Risk category-based analysis enforces the division of the whole dataset into smaller risk group portions, which means the correlations in risk groups are calculated based on a smaller sample size. The overall analysis gives a more holistic view of the language used by suicidal users, which can provide new correlations, lost in risk group-based analysis. Additionally, those reappearing in the overall analysis can be considered as the most valuable in general. Although we acknowledge that the fact that a correlation for a category pair reappeared in the overall analysis could be caused by differences in numbers of samples in the risk group-based analysis, namely, correlations from more populated groups are more likely to reappear in the overall analysis. Moreover, the overall analysis also confirms the validity of the risk group-based analysis, as most of the correlations that appeared in the overall analysis were with only one category, namely, Word Count, which is also not sufficiently informative compared to other more vocabulary-specific categories.

##### Correlations Confirmed from Risk Group-Based Analysis

There were two category pair correlations which reappeared in the overall analysis, namely “Death and Discrepancy” and “Death and Focusfuture”. This confirms that death-related vocabulary is pervasive in suicidal messages. However, in practice, both of those correlations are most often represented by two simple sentence examples (and their various modifications), which notoriously appear in suicidal messages, namely, “I should kill myself” (Death [kill] and Discrepancy [should]) and “I will kill myself” (Death [kill] and Focusfuture [will]).

Correlation between Death and Discrepancy was the strongest correlated category pair in the overall analysis for True Positives (actual suicidal messages). This suggests that it can be considered a strong predictor (Table 44). The reason for it being so strong was that it also appears in High and Medium risk categories, which makes it a highly populated category pair. However, an important fact is that even comparing to High and Medium, the correlation is even stronger in All, which suggests that it also appears to some extent in other group risks but is lost among other correlations due to high cut-off threshold (see Section 4.3.2).

The second correlation was between Death and Focusfuture, which also appears in High. Here, however, the correlation is weaker, which suggests that there were not many additional cases for this correlation in other risk groups, which would strengthen the overall correlation (Table 45). Still, together with Discrepancy, Focusfuture makes up one of the most pervasive category pair, and as such can be consider as a valuable predictor.

##### New Correlations Not Appearing in Risk Group-Based Analysis

The only newly appearing Pearson correlations were between Word count and various subcategories of Psychological Processes (Anxiety, Sadness, Feel, Risk, Ingestion, Body) and Personal Concerns (Health, Home), and were all negative predictors, or disambiguators, meaning that the correlations were stronger for False Positives (messages that look like suicidal but are not) (Table 46). All correlations for FP were also positive, which would suggest that if someone is talking about their concerns and problems related to psychological processes, then the longer the message is, the less likely they would be to commit suicide. This can be backed by situations where the user writes the message to relieve stress or seeks help with solving such problems. However, practice shows that long messages containing such expressions of concern are often explanations of whole life stories, which can represent a farewell letter of actual suicidal user, and thus should be taken with caution, especially, since Spearman correlations do not confirm the tendencies in all cases.

##### Rejected Correlations

Pronouns and Rewards correlate more strongly in FPs in suicidal subreddits, however, we were not able to find any meaningful, coherent, and true relationships in the use of those two categories (Table 47).

Exclamations and Affect seem to correlate more strongly for FP in FPs from the suicidal risk group (Table 48). However, Affective processes (happy, cried, etc.) typically occur in sentences where the author is expressing their sincere emotions, which when conducted with high intensity, also often coincides with exclamations marks. Therefore, as this correlation is too general, and could occur in any circumstances, with no particular sentence pattern, we decided to not consider it as a predictor.

One of the potential correlations meaningful which for which there was a strong difference between TP correlations and FP correlations was the category pair of Apostrophes (“, ”, ‘, ’) and Personal concerns (topics related to things which people could consider as personal concerns, wuch as Work, Leisure, Home, Money, Religion, Death) in High risk group (Table 49).

Since this is a general category, one could expect a similar difference between Apostro and one of the more specific subcategories within Personal concerns, such as Death. And indeed this was the case (see in paragraph High above).

The difference between the correlations for Apostro_persconc was large (0.41277), so if at least one of the correlations was also strong (≥0.4 or ≤−0.4), we could consider searching for deeper meaning in this result. However, since both correlations, although statistically significant, were weak (TP = −0.16251, FP = 0.25026), for TP it was slightly negative, for FP slightly positive, but we cannot expect anything meaningful from such correlations. I.e., no specific characteristic can be expected for neither TP nor FP in this case. Since the difference of correlations for Spearman was negligible, this potential correlation was rejected.

Two categories which were potentially strong candidates for predictors were Focusfuture and Time orientated language in High (Table 50). There is a realistic probability for those two categories to correlate simply because TimeOrient is a super-category for Focusfuture (may, will, soon), with two other categories including: Past focus/focuspast (ago, did, talked), and Present focus/focuspresent (today, is, now). However, a strong postitive correlation for those categories in TP (0.54688 ****) with weak in FP (0.21270 ****) suggests, that users tend to speak more about the future in actual suicidal texts.

This tendency was confirmed for Spearman (TP = 0.32289 ****, FP = 0.20906 ****). This further confirms the relationship between the two categories, but since one is a subcategory of the other it is too obvious and thus not too informative.

There was a noticeable significant moderate correlation in TP between Money (audit, cash, owe) and Negations (no, not, never) (Table 51). However, in practice, it typically included phrases like “isn’t worth” (as in “Life isn’t worth living”), which, although being a proper suicidal phrase, the frequent metaphorical use of money-related terms did not allow for considering this category pair as a good predictor for suicidal texts.

Although there is a sufficiently strong correlation for Exclamation markers and Question markers, for both Pearson and Spearman in TP, it is dubious if correlations for such meaning-heavy contents as suicidal texts should be considered for punctuation only (Table 52).

Although there is a significant moderate correlation for Emoji and emoticons with Colons for FP, we can consider it a trivial correlation as there are many emoticons containing colons (“:-)”); thus, this correlation is rejected (Table 53).

Similarly to Emoji, Colons also correlate with URLs, which is obvious as colons typically appear in URL (“http://www…”), thus this correlation can be safely rejected (Table 54 and Table 55).

## 5. Discussion

### 5.1. Summary of Most Important Insights

The pragmalinguistic analysis of vocabulary used by Reddit users allowed for collecting a number of insights, some of which confirm the present knowledge base of suicidal language, while others were new, sometimes unexpected. The insights included both predictors, as well as disambiguators (category pairs suggesting that the user is not suicidal, or negative predictors) for messages potentially written by suicidal individuals.

Most importantly, differently from many previous studies, which focused on single category predictors, we focused on category pair predictors with additional normalization by non-suicidal messages, which reduces the possibility of a false predictor.

Some of the most prevalent and expressed single category suicidal predictors were Death-related words, as expected in suicidal posts. However, thanks to the category pair disambiguation, we were able to specify that death only becomes a predictor when it co-occurs with such other categories as Future-focused language, Informal language, Discrepancy, or 1st person pronouns (singular and plural). Especially Discrepancy was expressed often in various risk groups of subreddits (both high- and medium-risk groups).

Interestingly, thanks to the category-pair-based analysis, we were also able to specify situations where Death alone would become a false predictor, for example, when it co-occurs with Apostrophes, especially in high-risk subreddits.

In addition, Positive emotions and Rewards were one of the most expressed category pair predictors, which confirms present knowledge on suicidal texts, as suicidal individuals often welcome the upcoming event with relief after a long life full of tragic events and trauma.

Interestingly, Negative emotions, such as Anger, can also become predictors when they are accompanied by Exclamations. On the other hand, when Anger co-occurs with Biological processes, Health, and 1st person pronouns, it is a disambiguator, thus would become a false predictor if considered as a separate category.

Biological processes especially often appear in pair with other categories (Anger, Affect, 1st person pronouns) as disambiguators, but can also act as predictors with such category as Money, which usually refers to the user metaphorically expressing that their life is not worth living. This is also confirmed when Money is accompanied by the Health category (which to some extent overlaps with Bio).

As for the unexpected predictors, there was an unusual, yet confirmed in data, valid correlation for Colons with Exclamations and Dashes. Although this could be specific to the medium (Reddit posts), it does suggest a more general pattern, which could occur often especially on social media and other Computer-mediated Communication venues (direct messenger apps, blogs, etc.).

On the other hand, emojis often acted as disambiguators, especially when used together with other categories suggesting relaxation and confidence, such as Netspeak and Fillers. However, it should be noted, that Netspeak alone could also become a predictor, when used with Motion-related vocabulary.

### 5.2. Limitations of This Study

There were several limitations of this study we plan to address in the near future.

Firstly, we address the methodological limitations. One of the unavoidable limitation in any study of a similar kind to ours is the sample size limitation. Although the dataset we collected and analysed is at present the largest dataset of this kind, there is always a chance that different results would be obtained on a completely different sample from the same source (Reddit). It is also possible that data collected from a completely different source, such as Twitter, Facebook, or blog posts, would show different tendencies. Such tendencies could be a result of communication channel limitations enforced by the SNS platform (e.g., Twitter has a 280 character limitation, while Reddit posts, especially those of suicidal users explaining their decisions, tend to be very long, even exceeding 500 words), but also, could reveal a correction to the tendencies found in our data. Therefore, we plan to remedy the sample size limitation in the following way. We will collect and analyse more data from the same set of subreddits and annotate them with the same annotation experts as used in this study (for details see Section 3.3). However, as expert annotation is costly and time-consuming we will improve the efficiency of annotation by developing machine learning (ML) methods to annotate the data. ML methods are reliable for annotating obvious and simple examples, yet tend to make mistakes for borderline cases. In our future research, we will test the efficacy of various ML methods, specify the types of examples they handle sufficiently well and allow the ML classifiers to automatically annotate the easy examples, while the difficult examples will be annotated by experts.

Another methodological limitation in this study was in the proposed method for specifying relevant categories. All of the previous research similar to ours applied LIWC categories in a straightforward way (see Section 2 and Section 4.3.1 for details), and did not account for any possible mistakes LIWC could make. Therefore, there has been no previous method for normalization of LIWC errors and specifying relevant categories we could compare to. Although the proposed method allowed for discarding most of irrelevant LIWC category pair correlations, in those that remained some irrelevant categories still slipped through (Section 4.3.3), and even in the relevant categories we were still able to find irrelevant examples. To remedy this problem we plan the following steps. We will test various thresholds of the relevant category pair selection procedure (see Algorithm 1) to set an optimal trade-off between the coverage and precision of the method. The overall precision of the procedure can be estimated as the number of category pairs selected as relevant to the overall number of categories selected using the method. The method has an estimated overall precision of 0.8 (see Table 56). This means that when using it, it is still necessary to manually confirm the selected category pairs. As the procedure itself is conceptually coherent, the errors are the result of intermediary tools used to calculate the correlation between the categories, namely, LIWC. We noticed multiple times that since the scores provided by LIWC are based on simple string matching with a set of dictionaries, meaning there is no context or figurative expression processing taken into account, the scores attached to many examples do not represent any reliable correlation but are either a result of a chance, are data-dependent, or are simply a mistake occurring due to the simplicity of the tool. For example, in sentences, such as “My life is not worth living”, LIWC annotates categories such as Biological processes (“living”) or Health (“life”) and Money (“worth”), suggesting there is a correlation between those categories. However, LIWC uses the Money category explicitly and does not differentiate between metaphorical use of language, thus making this an erroneous suggestion in this case. Therefore, in our future research, we aim at improving the relevant category pair correlation selection procedure by improving or exchanging LIWC with a better tool. This could be conducted by adding an additional ML layer on LIWC differentiating between literary and metaphorical use of language. However, as recent developments in Natural Language Processing show promising results [108], we plan to build a ML-based system for annotation of such categories from scratch.

### 5.3. Ethical Considerations and Release Information

In this research, we followed ethical recommendations of Shing et al. (2018) [39] and Benton et al. (2017) [109]. Especially the latter suggests, that although research applying publicly available data, such as Reddit posts in this research, can be exempted from being reviewed by ethical review boards, it is still desirable that studies on health-related topics undergo such a review to assure nothing is overlooked from the researchers’ side. Therefore, the research we report in this paper was also approved by the Ethical Review Board of Kitami Institute of Technology.

Regarding the release of the dataset, we follow and improve on the guidelines by [39]. At this point, we do not plan a full open-source release. Any provision of the dataset will need to be followed by two layers of review. Primarily, the researchers interested in using this dataset in their research will need to obtain approval from their institution’s ethical review board or equivalent body. The submission will also need to be reviewed and approved by our board. Additionally, depending on each case, the dataset will be provided after adding an additional layer of anonymization, which includes removal and normalization of user names and private names as well as other private information such as age or address reveal. The provision will be allowed under a specific written agreement, and the research progress will be strictly monitored by Samurai Labs.

## 6. Conclusions and Future Directions

In this paper, we studied language used by suicidal users on Reddit social media platform. We firstly collected and annotated with experts and highly trained annotators a large-scale Reddit Suicidal Query dataset. Then we analysed the dataset in various ways, including the analysis of user activity before and after posting a suicidal message, and a pragmalinguistic analysis of vocabulary used by suicidal users. In the linguistic analysis, we applied LIWC, a dictionary-based toolset widely used in psychology and linguistic research. However, although LIWC provides a wide range of linguistic category annotations on text, the raw LIWC scores are not sufficiently reliable, or informative, Therefore, we proposed a procedure to discard unreliable and misleading LIWC categories. The in-depth analysis revealed that the procedure is 80% effective. Although this could be considered as sufficiently high, especially, since the categories are also confirmed later by human experts, the remaining 20% could lead to undesirable misleading conclusions which could result in building up a false knowledge base. As this is unacceptable in health-related research topics, especially in suicide-related studies, we conclude, that LIWC is not a recommended tool for such studies, as it performs only a simplistic raw word matching, which often leads to the annotation of unrelated categories. Therefore, in our future studies, we plan to propose a set of machine learning (ML) classifiers allowing the context-aware and more robust (e.g., not dictionary-based) annotation of the categories listed in LIWC.

Regardless of the disadvantages of LIWC found in this research, the analysis of the obtained results revealed a number of valuable insights regarding the vocabulary used by suicidal users in comparison to non-suicidal users. Some of those insights, such as frequent appearance of Death-related words in suicidal texts, confirm the present knowledge base of how suicidal individuals use language, yet others (co-occurrence of Colons with Exclamations), were new and unexpected.

Apart from using machine learning to annotate LIWC-like categories on text, we also plan to use the collected dataset to train a separate set of ML classifiers to distinguish between truly suicidal and non-suicidal posts. Modern society has inherently adapted to actively use social media on a daily basis, and the amount of information flooding the internet exceeds any amounts allowing a realistic purely human-based health monitoring. Therefore, it has become apparent that technology-supported health monitoring is becoming a necessity, especially, if the problem of suicide—globally one of the most prevalent causes of death, especially in the youth—is to be tackled with a sufficiently robust coverage [110,111]. Other major global causes of death, such as cancer, or heart diseases already welcomed technology support in the form of disease trace detection in patients’ X-ray images, etc. With the assurance of proper use by experts (clinicians, etc.), it is possible to utilize the predictive and pattern-recognition capabilities of technology, especially based on Machine Learning and more broadly—Artificial Intelligence. As highlighted by Shing et al. (2018) [39], “physiological evidence like tumors or seizures, psychiatric diagnosis is largely a pattern recognition task performed by clinicians”. Moreover, initial attempts to automatically detect suicidal contents has recently been proposed by Coppersmith et al. (2015) [37], Oh et al. (2017) [112], Walsh et al. (2017) [113], Shing et al. (2018) [39], Zirikly et al. (2019) [40], Ji et al. (2018) [100], Mesfin et al. (2020) [83], Ghosh et al. (2020) [85], Schoene et al. (2021) [101], and a few others. With the presented work we wish to further contribute to such research and help clinicians and psychologists quickly reach potential suicidal users which could allow for more efficient help, and, in effect—saving more lives.

## Figures and Tables

**Table 1:** Statistics of the data included in the dataset.

Item	Subset	TP	FP
# of posts	Suicidal	3640	1721
	High	586	856
	Medium	1466	2374
	Low (non-gaming)	638	4192
	Low (gaming)		2055
	Sum	6330	11,198
# of unique users	Suicidal	3304	1639
	High	529	754
	Medium	1380	2234
	Low (non-gaming)	554	3586
	Low (gaming)		1961
# of posts per user (average)	Suicidal	1.10	1.05
	High	1.11	1.14
	Medium	1.06	1.06
	Low (non-gaming)	1.15	1.17
	Low (gaming)		1.05
# of words	Suicidal	727,744	395,664
	High	143,987	230,762
	Medium	430,374	743,548
	Low (non-gaming)	101,145	666,867
	Low (gaming)		373,430
# of words per post (average)	Suicidal	199.93	229.90
	High	245.71	269.58
	Medium	293.57	313.20
	Low (non-gaming)	158.53	159.08
	Low (gaming)		181.72
# of sentences	Suicidal	89,470	46,338
	High	16,984	26,261
	Medium	50,690	86,035
	Low (non-gaming)	12,104	76,446
	Low (gaming)		41,997
# of words per sentence (average)	Suicidal	10.43	10.43
	High	10.08	10.41
	Medium	9.76	10.23
	Low (non-gaming)	10.92	11.15
	Low (gaming)		11.99
# of characters per post (average)	Suicidal	982.47	1138.88
	High	1221.73	1355.16
	Medium	1447.87	1563.40
	Low (non-gaming)	779.20	794.73
	Low (gaming)		949.30
# of characters per sentence (average)	Suicidal	50.64	51.97
	High	49.86	51.77
	Medium	47.77	50.66
	Low (non-gaming)	53.00	54.58
	Low (gaming)		61.34

**Table 2:** Results of the manual verification of user activity.

	Plan/Method + Farwell	Suicidal Thoughts + Cry for Help
**Still posting + last not suicidal**	12.70%	30.33%
**Still posting + last suicidal**	1.11%	10.66%
**Not posting + last not suicidal**	1.59%	3.28%
**Not posting + last suicidal**	22.22%	18.85%
**Deleted users + deleted post**	49.21%	33.61%
**User deleted + post exists**	3.17%	3.28%

**Table 3:** User activity distribution among TPs.

TPs	Percentage
**Posted after match**	78.28%
**Did not post after match**	7.24%
**Deleted**	12.42%

**Table 4:** User activity distribution among FPs.

FPs	Percentage
**Posted after match**	82.32%
**Did not post after match**	4.13%
**Deleted**	10.49%

**Table 5:** User activity breakdown for critical speech acts.

Individual Speech Acts per Speech Act Percentage			
	Plan/Method	Declaration	Suicidal Thoughts
**Total**	115	599	1402
**Posted after match**	68.70%	74.96%	79.39%
**1–5 Posts after match**	12.17%	9.68%	11.20%
**6–10 Posts after match**	5.22%	6.01%	5.06%
**11–15 Posts after match**	5.22%	2.50%	4.07%
**16+ Posts after match**	46.09%	56.76%	59.06%
**Did not post after match**	6.96%	5.84%	6.49%
**Deleted**	18.26%	15.69%	11.70%

**Table 6:** Distribution of user activity among combinations of the supporting speech acts Being Burden, Cry for Help, Farewell and Self-harm with critical speech acts.

Pairs with Plan/Method, per pair percentage				
	PM+BeingBurden	PM+CryForHelp	PM+Farewell	PM+SelfHarm
**Total**	13	9	45	5
**Posted after match**	69.23%	77.78%	66.67%	40.00%
**1–5 Posts after match**	38.46%	22.22%	4.44%	0.00%
**6–10 Posts after match**	7.69%	22.22%	2.22%	0.00%
**11–15 Posts after match**	0.00%	0.00%	0.00%	0.00%
**16+ Posts after match**	23.08%	33.33%	60.00%	40.00%
**Did not post after match**	23.08%	0.00%	8.89%	20.00%
**Deleted**	7.69%	22.22%	17.78%	40.00%
**Pairs with Declaration. per pair percentage**				
	**D+BeingBurden**	**D+CryForHelp**	**D+Farewell**	**D+SelfHarm**
**Total**	82	55	20	49
**Posted after match**	80.49%	80.00%	50.00%	79.59%
**1–5 Posts after match**	6.10%	12.73%	0.00%	14.29%
**6–10 Posts after match**	2.44%	5.45%	0.00%	6.12%
**11–15 Posts after match**	4.88%	7.27%	0.00%	4.08%
**16+ Posts after match**	67.07%	54.55%	50.00%	55.10%
**Did not post after match**	10.98%	5.45%	35.00%	6.12%
**Deleted**	8.54%	10.91%	10.00%	10.20%
**Pairs with SuicidalThoughts. per pair percentage**				
	**ST+BeingBurden**	**ST+CryForHelp**	**ST+Farewell**	**ST+SelfHarm**
**Total**	219	160	20	171
**Posted after match**	79.91%	78.75%	80.00%	84.80%
**1–5 Posts after match**	14.61%	7.50%	0.00%	6.43%
**6–10 Posts after match**	8.22%	6.88%	0.00%	4.68%
**11–15 Posts after match**	4.57%	4.38%	0.00%	3.51%
**16+ Posts after match**	52.51%	60.00%	80.00%	70.18%
**Did not post after match**	10.05%	5.63%	5.00%	4.68%
**Deleted**	9.59%	14.38%	15.00%	8.77%
**Pairs with any Critical. per pair percentage**				
	**C+BeingBurden**	**C+CryForHelp**	**C+Farewell**	**C+SelfHarm**
**Total**	480	321	152	320
**Posted after match**	78.75%	81.31%	67.11%	83.75%
**1-5 Posts after match**	12.29%	10.59%	6.58%	9.38%
**6-10 Posts after match**	6.04%	5.30%	3.29%	5.31%
**11-15 Posts after match**	4.17%	4.67%	3.29%	3.75%
**16+ Posts after match**	56.25%	60.75%	53.95%	65.31%
**Did not post after match**	11.46%	4.98%	13.82%	6.25%
**Deleted**	9.38%	12.46%	15.13%	8.13%

**Table 7:** Average number of ‘posted after’ and ‘suicidal (as detected by our system) posted after’ messages for the overall numbers of TP and FP ‘posted after’ messages respectively.

Averages within ’Posted after’ Corpus Entries		
	TP	FP
All Posted After	288.45	613.85
Suicidal Posted After	3.34	1.56

**Table 8:** Positive emotions and Rewards in Suicidal.

Measure	Difference	Category Pair	TP Correlation	FP Correlation
Pearson	0.28372	posemo_reward	TP = 0.36566 ****	FP = 0.08194 ***
Spearman	0.10173	posemo_reward	TP = 0.29415 ****	FP = 0.19242 ****

**Table 9:** Emojis and Netspeak in Suicidal.

Measure	Difference	Category Pair	TP Correlation	FP Correlation
Pearson	0.22333	emojis_netspeak	TP = 0.00703	FP = 0.23036 ****
Spearman	0.00496	emojis_netspeak	TP = 0.04314 **	FP = 0.04810 *

**Table 10:** Emojis and Fillers in Suicidal.

Measure	Difference	Category Pair	TP Correlation	FP Correlation
Pearson	0.22249	emojis_filler	TP = −0.0092	FP = 0.21329 ****
Spearman	0.0189	emojis_filler	TP = 0.0019	FP = 0.0208

**Table 11:** Motion and Netspeak in Suicidal.

Measure	Difference	Category Pair	TP Correlation	FP Correlation
Pearson	0.21728	motion_netspeak	TP = 0.33322 ****	FP = 0.11594 ****
Spearman	0.0001	motion_netspeak	TP = 0.11849 ****	FP = 0.11839 ****

**Table 12:** Colons and Exclamations in Suicidal.

Measure	Difference	Category Pair	TP Correlation	FP Correlation
Pearson	0.20916	Colon_Exclam	TP = 0.22523 ****	FP = 0.01607
Spearman	0.04653	Colon_Exclam	TP = 0.12120 ****	FP = 0.07467 **

**Table 13:** Authentic and Social in Suicidal.

Measure	Difference	Category Pair	TP Correlation	FP Correlation
Pearson	0.18818	authentic_social	TP = −0.19062 ****	FP = −0.37880 ****
Spearman	0.14063	authentic_social	TP = −0.22599 ****	FP = −0.36662 ****

**Table 14:** Exclamations and Negative emotions/Anger in Suicidal.

Measure	Difference	Category Pair	TP Correlation	FP Correlation
Pearson	0.18427	Exclam_negemo	TP = 0.005	FP = 0.18927 ****
	0.18235	Exclam_anger	TP = 0.01149	FP = 0.19384 ****
Spearman	0.02306	Exclam_negemo	TP = 0.00099	FP = −0.02207
	0.00554	Exclam_anger	TP = 0.02947	FP = 0.03501

**Table 15:** Death and 1st person singular in Suicidal.

Measure	Difference	Category Pair	TP Correlation	FP Correlation
Pearson	0.11417	death_i	TP = 0.29340 ****	FP = 0.17923 ****
Spearman	0.1419	death_i	TP = 0.17024 ****	FP = 0.02834

**Table 16:** Death and Focusfuture in High.

Measure	Difference	Category Pair	TP Correlation	FP Correlation
Pearson	0.3882	death_focusfuture	TP = 0.42722 ****	FP = 0.03902
Spearman	0.04984	death_focusfuture	TP = −0.01599	FP = 0.03385

**Table 17:** Colon and Dash in High.

Measure	Difference	Category Pair	TP Correlation	FP Correlation
Pearson	0.38557	Colon_Dash	TP = 0.50178 ****	FP = 0.11621 ***
Spearman	0.01881	Colon_Dash	TP = 0.22793 ****	FP = 0.20912 ****

**Table 18:** Apostrophes and Death in High.

Measure	Difference	Category Pair	TP Correlation	FP Correlation
Pearson	0.36005	Apostro_death	TP = −0.00992	FP = 0.35013 ****
Spearman	0.0646	Apostro_death	TP = 0.03107	FP = −0.03353

**Table 19:** Periods and Death in High.

Measure	Difference	Category Pair	TP Correlation	FP Correlation
Pearson	0.35205	Period_death	TP = 0.27252 ****	FP = −0.07953 *
Spearman	0.05693	Period_death	TP = 0.11526 **	FP = 0.05833

**Table 20:** Ingest and Leisure in High.

Measure	Difference	Category Pair	TP Correlation	FP correlation
Pearson	0.35091	ingest_leisure	TP = 0.55548 ****	FP = 0.20457 ****
Spearman	0.02789	ingest_leisure	TP = 0.35925 ****	FP = 0.33136 ****

**Table 21:** Death and Informal language in High.

Measure	Difference	Category Pair	TP Correlation	FP Correlation
Pearson	0.33516	death_informal	TP = 0.44829 ****	FP = 0.11313 ***
	0.00863	death_swear	TP = 0.15865 ***	FP = 0.16728 ****
	0.03169	death_netspeak	TP = 0.00678	FP = −0.02491
	0.0376	assent_death	TP = −0.04639	FP = −0.00879
	0.04839	death_nonflu	TP = −0.07932	FP = −0.03093
	0.02615	death_filler	TP = −0.02165	FP = −0.0478
Spearman	0.06354	death_informal	TP = 0.03545	FP = −0.02809

**Table 22:** Death and Discrepancy in High.

Measure	Difference	Category Pair	TP Correlation	FP correlation
Pearson	0.28641	death_discrep	TP = 0.51509 ****	FP = 0.22868 ****
Spearman	0.16371	death_discrep	TP = 0.13673 ***	FP = −0.02698

**Table 23:** Death and Word count in High.

Measure	Difference	Category Pair	TP Correlation	FP Correlation
Spearman	0.42125	death_wc	TP = −0.57660 ****	FP = −0.15535 ****
Pearson	0.19399	death_wc	TP = −0.35897 ****	FP = −0.16498 ****

**Table 24:** Colons and Exclamation marks in High.

Measure	Difference	Category Pair	TP Correlation	FP correlation
Spearman	0.29528	Colon_Exclam	TP = 0.03088	FP = 0.32616 ****
Pearson	0.07493	Colon_Exclam	TP = 0.09807 *	FP = 0.17300 ****

**Table 25:** Death and 1st person plural in High.

Measure	Difference	Category Pair	TP Correlation	FP Correlation
Spearman	0.26507	death_we	TP = −0.30462 ****	FP = −0.03955
Pearson	0.04912	death_we	TP = −0.11842 **	FP = −0.06930 *

**Table 26:** Death and 3rd person singular in High.

Measure	Difference	Category Pair	TP Correlation	FP Correlation
Spearman	0.21201	death_shehe	TP = −0.28296 ****	FP = −0.07095 *
Pearson	0.0746	death_shehe	TP = −0.15761 ***	FP = −0.08301 *

**Table 27:** Affect and Bio in Medium.

Measure	Difference	Category Pair	TP Correlation	FP Correlation
Pearson	0.24465	affect_bio	TP = 0.16563 ****	FP = 0.41028 ****
Spearman	0.0116	affect_bio	TP = 0.16222 ****	FP = 0.15062 ****

**Table 28:** Anger and Bio in Medium.

Measure	Difference	Category Pair	TP Correlation	FP Correlation
Pearson	0.26287	anger_bio	TP = 0.29268 ****	FP = 0.55555 ****
Spearman	0.02161	anger_bio	TP = 0.21218 ****	FP = 0.19057 ****

**Table 29:** Anger and Health in Medium.

Measure	Difference	Category Pair	TP Correlation	FP Correlation
Pearson	0.33078	anger_health	TP = 0.0099	FP = 0.34068 ****
Spearman	0.01929	anger_health	TP = −0.0263	FP = −0.04559 *

**Table 30:** Anger and 1st person pronoun in Medium.

Measure	Difference	Category Pair	TP Correlation	FP Correlation
Pearson	0.2428	anger_i	TP = 0.16422 ****	FP = 0.40702 ****
Spearman	0.00564	anger_i	TP = 0.09066 ***	FP = 0.09630 ****

**Table 31:** Biological processes and Negative emotions in Medium.

Measure	Difference	Category Pair	TP Correlation	FP Correlation
Pearson	0.24471	bio_negemo	TP = 0.23695 ****	FP = 0.48166 ****
Spearman	0.01143	bio_negemo	TP = 0.18781 ****	FP = 0.19924 ****

**Table 32:** Death and Discrepancy in Medium.

Measure	Difference	Category Pair	TP Correlation	FP Correlation
Pearson	0.26624	death_discrep	TP = 0.68571 ****	FP = 0.41947 ****
Spearman	0.17214	death_discrep	TP = 0.23934 ****	FP = 0.06720 **

**Table 33:** 1st person pronoun and Sexual words in Medium.

Measure	Difference	Category Pair	TP Correlation	FP Correlation
Pearson	0.27041	i_sexual	TP = −0.03669	FP = 0.23372 ****
Spearman	0.09646	i_sexual	TP = −0.11728 ****	FP = −0.02082

**Table 34:** Personal pronouns and Sexual words in Medium.

Measure	Difference	Category Pair	TP Correlation	FP Correlation
Pearson	0.2341	ppron_sexual	TP = −0.02727	FP = 0.20683 ****
Spearman	0.11721	ppron_sexual	TP = −0.0392	FP = 0.07801 ***

**Table 35:** Health and Money in Low.

Measure	Difference	Category Pair	TP Correlation	FP Correlation
Pearson	0.59047	health_money	TP = 0.54485 ****	FP = −0.04562 ***
Spearman	0.01536	health_money	TP = 0.13343 ***	FP = 0.11807 ****

**Table 36:** Bio and Money in Low.

Measure	Difference	Category Pair	TP Correlation	FP Correlation
Pearson	0.46141	bio_money	TP = 0.39224 ****	FP = −0.06917 ****
Spearman	0.06638	bio_money	TP = 0.07078	FP = 0.0044

**Table 37:** Filler and Informal language in Low.

Measure	Difference	Category Pair	TP Correlation	FP Correlation
Pearson	0.45521	filler_informal	TP = 0.57890 ****	FP = 0.12369 ****
Spearman	0.12826	filler_informal	TP = 0.30792 ****	FP = 0.17966 ****

**Table 38:** Filler and Non-fluency in Low.

Measure	Difference	Category Pair	TP Correlation	FP Correlation
Pearson	0.41115	filler_nonflu	TP = 0.40190 ****	FP = −0.00925
Spearman	0.09675	filler_nonflu	TP = 0.19495 ****	FP = 0.09820 ****

**Table 39:** Body and Power in Low.

Measure	Difference	Category Pair	TP Correlation	FP Correlation
Pearson	0.26776	body_power	TP = 0.30840 ****	FP = 0.04064 **
Spearman	0.03902	body_power	TP = 0.17674 ****	FP = 0.13772 ****

**Table 40:** Adjectives and Swearing in Low.

Measure	Difference	Category Pair	TP Correlation	FP Correlation
Pearson	0.25332	adj_swear	TP = 0.43153 ****	FP = 0.17821 ****
Spearman	0.11019	adj_swear	TP = 0.26580 ****	FP = 0.15561 ****

**Table 41:** Adjectives and Sexual in Low.

Measure	Difference	Category Pair	TP Correlation	FP Correlation
Pearson	0.22683	adj_sexual	TP = 0.43184 ****	FP = 0.20501 ****
Spearman	0.06348	adj_sexual	TP = 0.26226 ****	FP = 0.19878 ****

**Table 42:** Anger and Bio in Low.

Measure	Difference	Category Pair	TP Correlation	FP Correlation
Pearson	0.22315	anger_bio	TP = 0.29412 ****	FP = 0.51727 ****
Spearman	0.0113	anger_bio	TP = 0.26006 ****	FP = 0.27136 ****

**Table 43:** Home and Leisure in Low.

Measure	Difference	Category Pair	TP Correlation	FP Correlation
Spearman	0.30123	home_leisure	TP = 0.42936 ****	FP = 0.12813 ****
Pearson	0.25261	home_leisure	TP = 0.23557 ****	FP = −0.01704

**Table 44:** Death and Discrepancy in All.

Measure	Difference	Category Pair	TP Correlation	FP Correlation
Pearson	0.46274	death_discrep	TP = 0.44827 ****	FP = −0.01447
Spearman	0.21449	death_discrep	TP = 0.20002 ****	FP = −0.01447

**Table 45:** Death and Focusfuture in All.

Measure	Difference	Category Pair	TP Correlation	FP Correlation
Pearson	0.36127	death_focusfuture	TP = 0.35304 ****	FP = −0.00823
Spearman	0.06345	death_focusfuture	TP = 0.05522 ****	FP = −0.00823

**Table 46:** Word count and Anxiety, Health, Sadness, Risk, Feel, Home, Ingestion, Body in All.

Measure	Difference	Category Pair	TP Correlation	FP Correlation
Pearson	0.4349	anx_wc	TP = 0.00518	FP = 0.44008 ****
	0.41986	health_wc	TP = −0.10468 ****	FP = 0.31518 ****
	0.41832	sad_wc	TP = −0.07579 ****	FP = 0.34253 ****
	0.40824	risk_wc	TP = 0.00102	FP = 0.40926 ****
	0.39077	feel_wc	TP = −0.05136 ****	FP = 0.33941 ****
	0.38976	home_wc	TP = 0.04887 ***	FP = 0.43863 ****
	0.37051	ingest_wc	TP = 0.01514	FP = 0.38565 ****
	0.35121	body_wc	TP = −0.02784 *	FP = 0.32337 ****
Spearman	0.16257	anx_wc	TP = 0.27751 ****	FP = 0.44008 ****
	0.30037	health_wc	TP = 0.01481	FP = 0.31518 ****
	0.24418	sad_wc	TP = 0.09835 ****	FP = 0.34253 ****
	0.17481	risk_wc	TP = 0.23445 ****	FP = 0.40926 ****
	0.19291	feel_wc	TP = 0.14650 ****	FP = 0.33941 ****
	0.10963	home_wc	TP = 0.32900 ****	FP = 0.43863 ****
	0.06737	ingest_wc	TP = 0.31828 ****	FP = 0.38565 ****
	0.08753	body_wc	TP = 0.23584 ****	FP = 0.32337 ****

**Table 47:** Pronouns and Rewards in Suicidal.

Measure	Difference	Category Pair	TP Correlation	FP Correlation
Pearson	0.20745	pronoun_reward	TP = −0.06580 ****	FP = 0.14165 ****
Spearman	0.01419	pronoun_reward	TP = −0.06760 ****	FP = −0.05341 *

**Table 48:** Exclamations and Affect in Suicidal.

Measure	Difference	Category Pair	TP Correlation	FP Correlation
Pearson	0.18891	Exclam_affect	TP = 0.02109	FP = 0.21000 ****
Spearman	0.00826	Exclam_affect	TP = 0.02523	FP = 0.01697

**Table 49:** Apostrophes and Personal concerns in High.

Measure	Difference	Category Pair	TP Correlation	FP Correlation
Pearson	0.41277	Apostro_persconc	TP = −0.16251 ****	FP = 0.25026 ****
Spearman	0.07903	Apostro_persconc	TP = −0.16649 ****	FP = −0.08746 *

**Table 50:** Focusfuture and Time orientated language in High.

Measure	Difference	Category Pair	TP Correlation	FP Correlation
Pearson	0.33418	focusfuture_timeorient	TP = 0.54688 ****	FP = 0.21270 ****
Spearman	0.11383	focusfuture_timeorient	TP = 0.32289 ****	FP = 0.20906 ****

**Table 51:** Money and Negations in Low.

Measure	Difference	Category Pair	TP Correlation	FP Correlation
Pearson	0.27499	money_negate	TP = 0.25119 ****	FP = −0.0238
Spearman	0.01516	money_negate	TP = 0.08205 *	FP = 0.09721 ****

**Table 52:** Exclamation Markers and Question Markers in Medium.

Measure	Difference	Category Pair	TP Correlation	FP Correlation
Pearson	0.28705	Exclam_QMark	TP = 0.31996 ****	FP = 0.03291
Spearman	0.07154	Exclam_QMark	TP = 0.20944 ****	FP = 0.13790 ****

**Table 53:** Colons and Emojis in Medium.

Measure	Difference	Category Pair	TP Correlation	FP Correlation
Pearson	0.33811	Colon_emojis	TP = −0.01616	FP = 0.32195 ****
Spearman	0.06389	Colon_emojis	TP = −0.03211	FP = 0.03178

**Table 54:** Colons and Urls in Suicidal.

Measure	Difference	Category Pair	TP Correlation	FP Correlation
Pearson	0.59949	Colon_urls	TP = 0.05197 **	FP = 0.65146 ****
Spearman	0.08488	Colon_urls	TP = 0.08886 ****	FP = 0.17374 ****

**Table 55:** Colons and URLs in Medium.

Measure	Difference	Category Pair	TP Correlation	FP Correlation
Pearson	0.31149	Colon_urls	TP = 0.07578 **	FP = 0.38727 ****
Spearman	0.01373	Colon_urls	TP = 0.08186 **	FP = 0.09559 ****

**Table 56:** Precision of relevant category pair correlation selection procedure.

	Relevant	Rejected	All	Precision
Suicidal	8	3	11	0.73
High	11	2	13	0.85
Medium	8	3	11	0.73
Low	9	1	10	0.90
Overall	36	9	45	0.80

## Data Availability

Due to delicate character if the data, it will not be made widely available. Any inquiries about the possibility of data availability should be send to Samurai Labs.

## Appendix A. Division of Subreddits into Risk Levels

The following appendix contains subreddits (as of August 2020) divided by their risk level defined by how frequently suicidal messages appear on them.

Suicidal Subreddits (highest risk):	/r/SuicideWatch/, /r/depression_help/, /r/depression/, /r/MentalHealthSupport/, /r/depressed/, /r/Suicidal_Thoughts/, /r/TeenageSuicideWatch/, /r/Deppression/, /r/Mental_Help/, /r/AdultSelfHarm/, /r/Suicide_help/, /r/iwanttokillmyself/, /r/ihatemylife/, /r/iwannadie/
High-risk Subreddits:	/r/benzorecovery/, /r/mentalillness/, /r/selfharm/, /r/mentalhealth/, /r/BPD/, /r/abusiveparents/, /r/BorderlinePDisorder/, /r/emotionalabuse/, /r/AnxietyDepression/, /r/SuicideBereavement/, /r/dysthymia/, /r/BPDsupport/, /r/SelfHate/, /r/TransVent/, /r/ineedhelp/, /r/Existential_crisis/, /r/DepressionRecovery/, /r/Borderline/
Medium-risk Subreddits:	/r/abuse/, /r/PMDD/, /r/widowers/, /r/virgin/, /r/ptsd/, /r/Vent/, /r/bipolar/, /r/fuckeatingdisorders/, /r/BodyDysmorphia/, /r/offmychest/, /r/LifeAdvice/, /r/BipolarReddit/, /r/venting/, /r/Anxietyhelp/, /r/sad/, /r/CPTSD/, /r/selfhelp/, /r/adultsurvivors/, /r/loveafterporn/, /r/raisedbynarcissists/, /r/lonely/, /r/dpdr/, /r/askatherapist/, /r/TalkTherapy/, /r/therapy/, /r/domesticviolence/, /r/narcissisticparents/, /r/KindVoice/, /r/AvPD/, /r/MomForAMinute/, /r/JUSTNOFAMILY/, /r/SeriousConversation/, /r/abusiverelationships/, /r/OCD/, /r/rapecounseling/, /r/MadeOfStyrofoam/, /r/Doomers/, /r/helpme/, /r/Psychosis/, /r/rape/, /r/bipolar2/, /r/Anxiety/, /r/confessions/, /r/TrueOffMyChest/, /r/ForeverAlone/, /r/HOCD/, /r/schizophrenia/, /r/HealthAnxiety/, /r/DysfunctionalFamily/, /r/StopSelfHarm/, /r/death/, /r/motherinlawsfromhell/, /r/Guilt/, /r/DiaryOfARedditor/, /r/PanicAttack/, /r/loneliness/, /r/prozac/, /r/MMFB/, /r/malementalhealth/, /r/survivorsofabuse/, /r/depressionregimens/, /r/Postpartum_Depression/, /r/EatingDisorderHope/, /r/BPD4BPD/, /r/transgender_support/, /r/psychopath/, /r/needhelp/, /r/ToxicRelationships/
Low-risk Subreddits:	Other

## Appendix B. Full List of LIWC Categories with Explanations

Full list of LIWC categories with explanations and examples used in this research. Table compiled on the basis of information included in Tausczik et al. (2010) [95] and Pennebaker et al. (2015) [96].

**Category**	**Label**	**Examples**		**Category**	**Label**	**Examples**
Word count	WC	–		…		
**Summary Language Variables**				**Psychological Processes (continuation)**		
Analytical thinking	Analytic	–		Cognitive processes	cogproc	cause, know, ought
Clout	Clout	–		Insight	insight	think, know
Authentic	Authentic	–		Causation	cause	because, effect
Emotional tone	Tone	–		Discrepancy	discrep	should, would
Words/sentence	WPS	–		Tentative	tentat	maybe, perhaps
Words > 6 letters	Sixltr	–		Certainty	certain	always, never
Dictionary words	Dic	–		Differentiation	differ	hasn’t, but, else
**Linguistic Dimensions**				Perceptual processes	percept	look, heard, feeling
Total function words	funct	it, to, no, very		See	see	view, saw, seen
Total pronouns	pronoun	I, them, itself		Hear	hear	listen, hearing
Personal pronouns	ppron	I, them, her		Feel	feel	feels, touch
1st pers singular	i	I, me, mine		Biological processes	bio	eat, blood, pain
1st pers plural	we	we, us, our		Body	body	cheek, hands, spit
2nd person	you	you, your, thou		Health	health	clinic, flu, pill
3rd pers singular	shehe	she, her, him		Sexual	sexual	horny, love, incest
3rd pers plural	they	they, their, they’d		Ingestion	ingest	dish, eat, pizza
Impersonal pronouns	ipron	it, it’s, those		Drives	drives	
Articles	article	a, an, the		Affiliation	affiliation	ally, friend, social
Prepositions	prep	to, with, above		Achievement	achieve	win, success, better
Auxiliary verbs	auxverb	am, will, have		Power	power	superior, bully
Common Adverbs	adverb	very, really		Reward	reward	take, prize, benefit
Conjunctions	conj	and, but, whereas		Risk	risk	danger, doubt
Negations	negate	no, not, never		Time orientations	TimeOrient	
**Other Grammar**				Past focus	focuspast	ago, did, talked
Common verbs	verb	eat, come, carry		Present focus	focuspresent	today, is, now
Common adjectives	adj	free, happy, long		Future focus	focusfuture	may, will, soon
Comparisons	compare	greater, best, after		Relativity	relativ	area, bend, exit
Interrogatives	interrog	how, when, what		Motion	motion	arrive, car, go
Numbers	number	second, thousand		Space	space	down, in, thin
Quantifiers	quant	few, many, much		Time	time	end, until, season
**Psychological Processes**				**Personal concerns**		
Affective processes	affect	happy, cried		Work	work	job, majors, xerox
Positive emotion	posemo	love, nice, sweet		Leisure	leisure	cook, chat, movie
Negative emotion	negemo	hurt, ugly, nasty		Home	home	kitchen, landlord
Anxiety	anx	worried, fearful		Money	money	audit, cash, owe
Anger	anger	hate, kill, annoyed		Religion	relig	altar, church
Sadness	sad	crying, grief, sad		Death	death	bury, coffin, kill
Social processes	social	mate, talk, they		Informal language	informal	
Family	family	daughter, dad, aunt		Swear words	swear	fuck, damn, shit
Friends	friend	buddy, neighbor		Netspeak	netspeak	btw, lol, thx
Female references	female	girl, her, mom		Assent	assent	agree, OK, yes
Male references	male	boy, his, dad		Nonfluencies	nonflu	er, hm, umm
…				Fillers	filler	Imean, youknow
